# Tris(4,4′-di-*tert*-butyl-2,2′-bipyridine-κ^2^ *N*,*N*′)molybdenum(II) μ_6_-oxido-dodeca-μ_2_-oxido-hexa­oxidohexa­molybdate(VI) acetonitrile tetra­solvate

**DOI:** 10.1107/S1600536811049385

**Published:** 2011-11-25

**Authors:** Tatiana R. Amarante, José A. Fernandes, Isabel S. Gonçalves, Filipe A. Almeida Paz

**Affiliations:** a Department of Chemistry, University of Aveiro, CICECO, 3810-193 Aveiro, Portugal

## Abstract

The asymmetric unit of the title compound, [Mo(C_18_H_24_N_2_)_3_][Mo_6_O_19_]·4CH_3_CN, comprises an [Mo(di-*t*-Bu-bipy)_3_]^2+^ cation (di-*t*-Bu-bipy is 4,4′-di-*tert*-butyl-2,2′-bipyridine), two halves of Lindqvist-type [Mo_6_O_19_]^2−^ anions (with each anion completed by the application of a center of inversion) and four acetonitrile solvent mol­ecules. The geometry around the metal atom of the cation resembles a distorted octa­hedron, with each of the three di-*t*-Bu-bipy ligands being almost planar [deviation from planarity < 6.3 (2)°]. Supra­molecular inter­actions, namely Mo=O⋯π, C N⋯π, C—H⋯O and C—H⋯N, along with electrostatic forces, mediate the crystal packing. Two of the *tert*-butyl groups are affected by rotational disorder which was modeled over two distinct positions with major site occupancies of 0.707 (9) and 0.769 (8).

## Related literature

For general literature on polyoxidometalates, see: Allcock *et al.* (1973 ▶); Long *et al.* (2007 ▶, 2010 ▶); Pope & Müller (1991 ▶). For examples of coordination compounds with the Lindqvist [Mo_6_O_19_]^2−^ anion, see: Burkholder & Zubieta (2004 ▶); Devi & Zubieta (2002 ▶); Fan *et al.* (2010 ▶); Liu *et al.* (2010 ▶); Sarma *et al.* (2011 ▶); Vrdoljak *et al.* (2011 ▶); Wang *et al.* (2005 ▶). For examples of compounds with the 2,2′-bipyridine ligand and derivatives, see: Abrantes *et al.* (2010 ▶); Amarante *et al.* (2009 ▶, 2010 ▶); Schwalbe *et al.* (2008 ▶). For a description of the Cambridge Structural Database, see: Allen (2002 ▶).
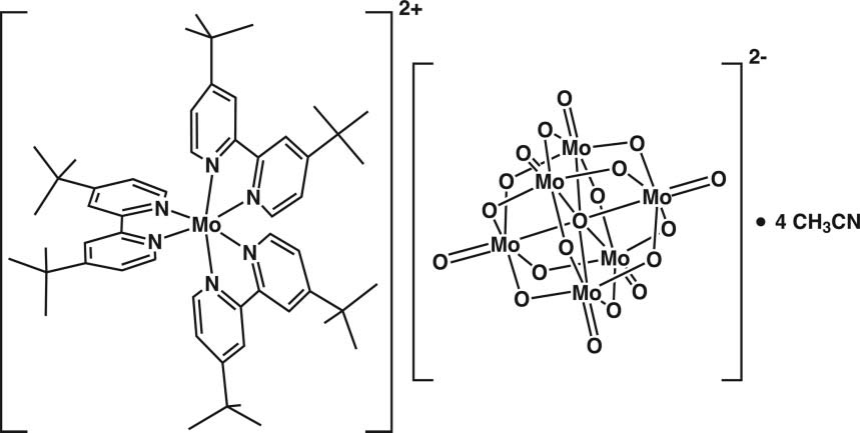


## Experimental

### 

#### Crystal data

[Mo(C_18_H_24_N_2_)_3_][Mo_6_O_19_]·4C_2_H_3_N
*M* *_r_* = 1944.97
Triclinic, 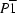

*a* = 14.4202 (8) Å
*b* = 16.3205 (9) Å
*c* = 17.1122 (10) Å
α = 90.144 (3)°
β = 103.862 (2)°
γ = 107.547 (2)°
*V* = 3715.9 (4) Å^3^
*Z* = 2
Mo *K*α radiation
μ = 1.22 mm^−1^
*T* = 150 K
0.17 × 0.12 × 0.08 mm

#### Data collection

Bruker X8 KappaCCD APEXII diffractometer
Absorption correction: multi-scan (*SADABS*; Sheldrick, 1997 ▶) *T*_min_ = 0.820, *T*_max_ = 0.909
209835 measured reflections
22527 independent reflections
17706 reflections with *I* > 2σ(*I*)
*R*_int_ = 0.039

#### Refinement

*R*[*F* ^2^ > 2σ(*F* ^2^)] = 0.048
*wR*(*F* ^2^) = 0.149
*S* = 1.06
22527 reflections
970 parameters
18 restraints
H-atom parameters constrained
Δρ_max_ = 2.29 e Å^−3^
Δρ_min_ = −3.96 e Å^−3^

### 

Data collection: *APEX2* (Bruker, 2006 ▶); cell refinement: *SAINT-Plus* (Bruker, 2005 ▶); data reduction: *SAINT-Plus*; program(s) used to solve structure: *SHELXTL* (Sheldrick, 2008 ▶); program(s) used to refine structure: *SHELXTL*; molecular graphics: *DIAMOND* (Brandenburg, 2009 ▶); software used to prepare material for publication: *SHELXTL*.

## Supplementary Material

Crystal structure: contains datablock(s) global, I. DOI: 10.1107/S1600536811049385/tk5024sup1.cif

Structure factors: contains datablock(s) I. DOI: 10.1107/S1600536811049385/tk5024Isup2.hkl

Additional supplementary materials:  crystallographic information; 3D view; checkCIF report

## Figures and Tables

**Table 1:** Selected bond lengths (Å)

Mo1—N1	2.117 (3)
Mo1—N2	2.113 (3)
Mo1—N3	2.090 (3)
Mo1—N4	2.113 (3)
Mo1—N5	2.138 (3)
Mo1—N6	2.103 (3)

**Table 2:** Selected short inter­actions (Å, °) *Cg*1, *Cg*2 and *Cg*3 are the centroids of the C1–C5, C6–C10 and C19–C23 rings, respectively.

*A*—*B*⋯*C*	*A*—*B*	*B*⋯*C*	*A*⋯*C*	<(*A*—*B*⋯*C*)
***Y*—*X*⋯π contacts**				
Mo4—O10⋯*Cg*1^i^	1.69 (1)	3.15 (1)	4.393 (2)	128 (1)
Mo5—O15⋯*Cg*2^ii^	1.69 (1)	3.40 (1)	4.622 (2)	128 (1)
C102—N101⋯*Cg*2	1.16 (1)	3.40 (1)	3.473 (8)	84 (1)
C102—N101⋯*Cg*3	1.16 (1)	3.56 (1)	3.762 (8)	91 (1)
**Weak hydrogen bonds**				
C16—H16*A*⋯N101^ii^	0.98	2.60	3.537 (14)	160
C19—H19⋯O10^i^	0.95	2.45	3.331 (5)	154
C27—H27⋯O17^ii^	0.95	2.57	3.059 (6)	113
C36—H36*A*⋯O8^iii^	0.98	2.55	3.501 (8)	164
C49—H49*C*⋯O6^iv^	0.98	2.59	3.557 (8)	170

Symmetry codes: (i) 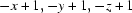; (ii) 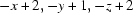; (iii) 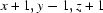; (iv) 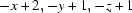.

**Fig. 1.**
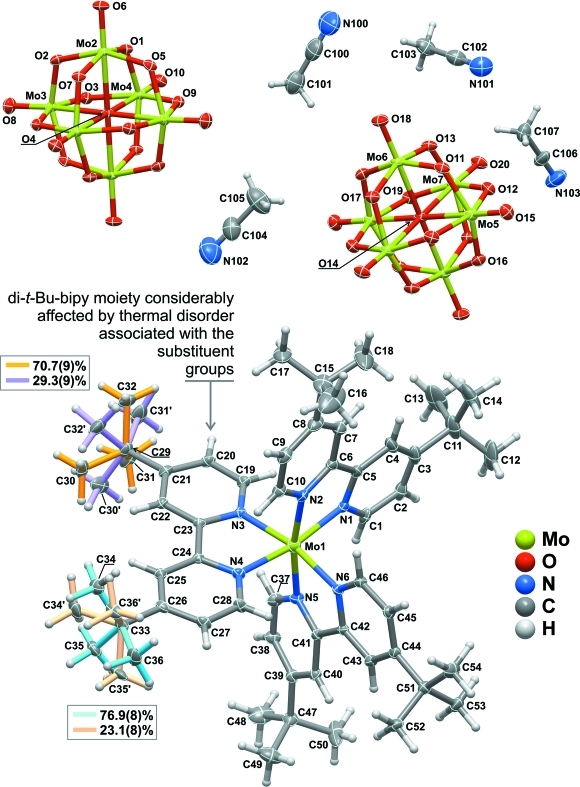
Schematic representation of the chemical species composing the asymmetric unit of the title compound. Displacement ellipsoids are drawn at the 30% probability level and the atomic labeling is provided for all non-hydrogen atoms belonging to the asymmetric unit. Hydrogen atoms are represented as small spheres with arbitrary radius. The rotational disorder associated with the tert-butyl moieties is depicted using different colors for each position. Symmetry operations used to complete the centrosymmetric POM anions: {Mo1—Mo4,O1—O10}: 1 - x, 2 - y, 1 - z; {Mo5—Mo8,O11—O20}: 1 - x, 1 - y, 2 - z.

**Fig. 2.**
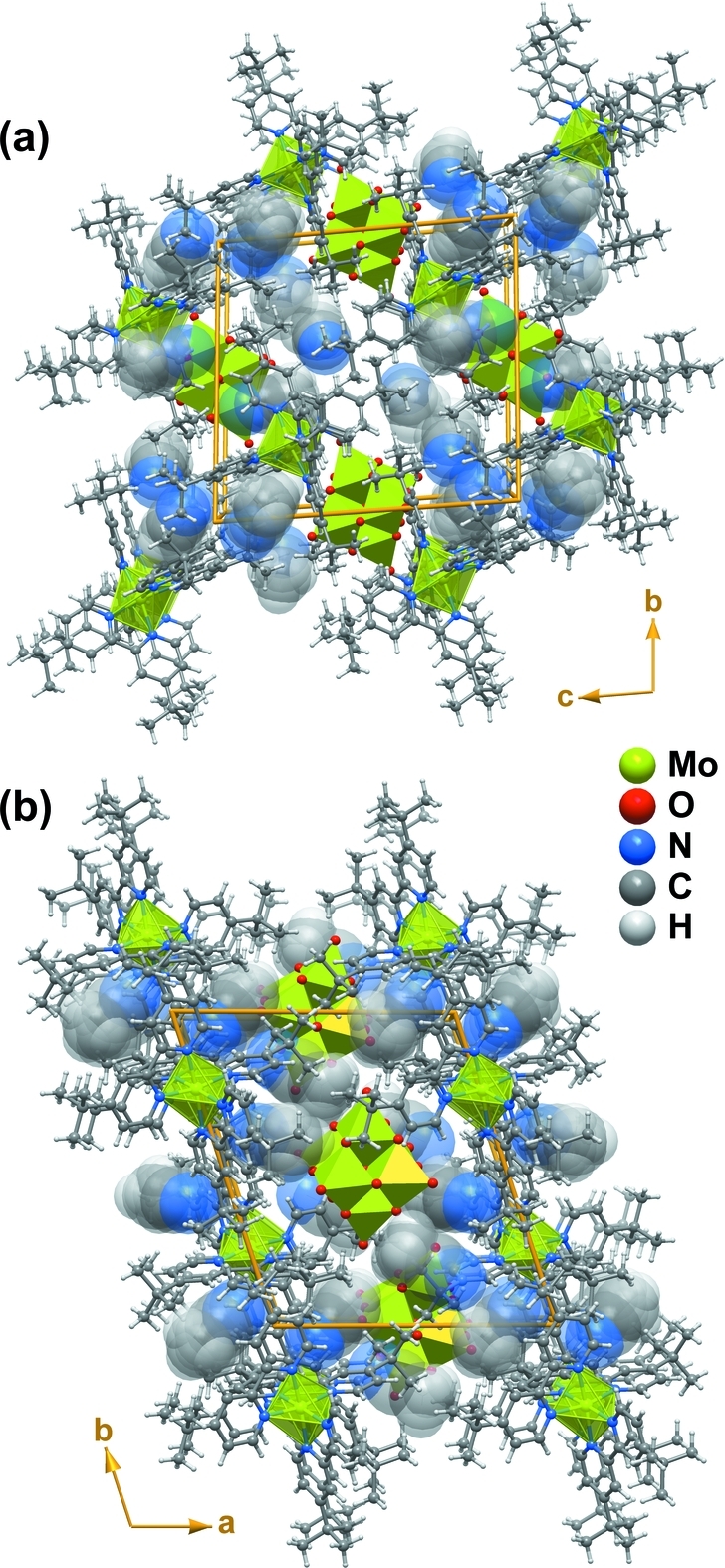
Crystal packing of the title compound viewed in perspective along the (a) [100] and (b) [001] directions of the unit cell. The {MoO6} and {MoN6} polyhedra are represented as green octahedra (opaque and translucent, respectively), and the acetonitrile molecules are represented in transparent space filling mode for clarity.

## supplementary crystallographic information

### Comment

Polyoxometalates (POM) are interesting compounds because of their structural and topological novelties as well as their optical, electronic, magnetic and catalytic properties (Pope & Müller, 1991; Long *et al.*, 2010). These chemical species are polynuclear oxyanions with variable sizes which may reach the nanometer scale. POMs have also been regarded as suitable anionic building units for organic-inorganic hybrid materials. A wide variety of hybrid POMs can be generated by hydrothermal synthesis or by standard benchtop methods (Long *et al.*, 2007). A search in the literature reveals that there is a wide variety of coordination compounds in which the Lindqvist [Mo_6_O_19_]^2-^ anion acts as counterion in crystal structures (Burkholder & Zubieta, 2004; Sarma *et al.*, 2011; Vrdoljak *et al.*, 2011). Among these known compounds only four contain bipyridine derivatives coordinated to metallic centers composing charge-balancing cations, namely [{Cu(4,4'-di-*tert*-butyl-2,2'-bipyridine)}Mo_6_O_19_] (Devi & Zubieta, 2002), [Co(2,2'-bipyridine)_3_]_2_[Mo_6_O_19_][β-(H_2_Mo_8_O_26_)]^.^4H_2_O (Wang *et al.*, 2005), [Co(2,2'-bipyridine)_3_]_2_[Mo_6_O_19_] (Liu *et al.*, 2010) and [Ni(2,2'-bipyridine)_3_][Mo_6_O_19_] (Fan *et al.*, 2010).

In our research group *N,N'*-chelating ligands, such as 2,2'-bipyridine and their derivatives, have been extensively employed in the preparation of oxomolybdenum compounds (Amarante *et al.*, 2009, 2010) and organic-inorganic hybrid materials (Abrantes *et al.*, 2010), to be subsequently applied in catalysis, especially in olefin epoxidation. Interestingly, while trying to recrystallize in acetonitrile the polynuclear complex [Mo_8_O_24_(di-*t*-Bu-bipy)_4_] (where di-*t*-Bu-bipy stands for 4,4'-di-*tert*-butyl-2,2'-bipyridine) (Amarante *et al.*, 2010), we unexpectedly isolated a single-crystal of the title compound whose crystal structure we wish to report.

The asymmetric unit consists of one [Mo(C_18_H_24_N_2_)_3_)]^2+^ cation {[Mo(di-*t*-Bu-bipy)_3_]^2+^}, two halves of crystallographically independent centrosymmetric Lindqvist-type [Mo_6_O_19_]^2-^ anions and four acetonitrile molecules as depicted in Fig. 1. The two crystallographically independent anions are located around centers of inversion of the triclinic space group *P*1 which are coincident with the central µ_6_-oxo atom of each moiety (O4 and O14 in Fig. 1). The geometrical features observed for these chemical moieties are typical (Allcock *et al.*, 1973) and will not be discussed any further in this crystallographic report. By contrast, the cation is to the best of our knowledge the second example of a coordination compound with general formula [*M*(di-*t*-Bu-bipy)_3_]^n+^, with the first example corresponding to a Ru^3+^ structure (Schwalbe *et al.*, 2008). The coordination geometry around Mo1 resembles a distorted octahedron with the Mo—N distances ranging from 2.090 (3) to 2.138 (3) Å. We note that these lengths are some of the shortest reported for a Mo—N distance, as revealed by a search in the Cambridge Structural Database for related compounds comprising molybdenum and 2,2'-bipyridine or its derivatives (Allen, 2002). The *cis* octahedral angles can be divided into two groups: while the bite angles related to the *N,N'*-chelating di-*t*-Bu-bipy range from 76.32 (12) to 77.40 (12)°, those involving two adjacent ligands range instead from 92.18 (12) to 97.09 (11)°. The *trans* octahedral angles were found in-between 167.87 (11) to 169.85 (12)°. The three crystallographically independent di-*t*-Bu-bipy ligands are almost planar, with the angles subtended by each pair of pyridine rings ranging from 1.41 (18) to 6.3 (2)°. In addition, the medium planes containing each di-*t*-Bu-bipy ligand are almost mutually perpendicular (angles ranging from 84.66 to 89.18 (11)°).

The crystal structure is rich in supramolecular contacts, among which some Mo═O···π, C≡N···π, C—H···O and C—H···N interactions are noteworthy (see Table 2 for details; interactions not shown). These contacts, along with the need to effectively fill the space mediated by electrostatic interactions, contribute to the crystal packing (Fig. 2).

### Experimental

The title compound was isolated during the recrystallization in acetonitrile of the polynuclear complex [Mo_8_O_24_(di-*t*-Bu-bipy)_4_] (1) (Amarante *et al.*, 2010). Crystals of 1 were harvested and the supernatant solution was partially evaporated in vacuum. After two days, pink crystals of the title compound suitable for X-ray diffraction analysis were obtained.

### Refinement

Hydrogen atoms bound to carbon were placed in idealized positions and were included in the final structural model in riding-motion approximation with C—H = 0.95 Å (aromatic C—H) and 0.98 Å (—CH_3_). The isotropic displacement parameters for these atoms were fixed at 1.2×*U*_eq_ (aromatic C—H) or 1.5×*U*_eq_ (—CH_3_) of the respective parent carbon atoms.

One di-*t*-Bu-bipy contains both *tert*-butyl groups highly disordered with the rotational displacement associated with the —CH_3_ moieties being modeled by the superposition of two parts (Fig. 1), whose occupancy was refined and, ultimately, converged to 0.231 (8): 0.769 (8) (for the C33 moiety), and 0.293 (9): 0.707 (9) (for the C29 moiety).

The largest peak and hole, 2.29 and -3.96 e^.^Å^-3^, are located at 0.70 Å from Mo6 and 0.36 Å from Mo1, respectively.

### Crystal data

**Table:**

[Mo(C_18_H_24_N_2_)_3_][Mo_6_O_19_]·4C_2_H_3_N	*Z* = 2
*M**_r_* = 1944.97	*F*(000) = 1944
Triclinic, *P*1	*D*_x_ = 1.738 Mg m^−^^3^
Hall symbol: -P 1	Mo *K*α radiation, λ = 0.71073 Å
*a* = 14.4202 (8) Å	Cell parameters from 9781 reflections
*b* = 16.3205 (9) Å	θ = 2.5–30.9°
*c* = 17.1122 (10) Å	µ = 1.22 mm^−^^1^
α = 90.144 (3)°	*T* = 150 K
β = 103.862 (2)°	Block, pink
γ = 107.547 (2)°	0.17 × 0.12 × 0.08 mm
*V* = 3715.9 (4) Å^3^	

### Data collection

**Table:**

Bruker X8 KappaCCD APEXII diffractometer	22527 independent reflections
Radiation source: fine-focus sealed tube	17706 reflections with *I* > 2σ(*I*)
graphite	*R*_int_ = 0.039
ω and φ scans	θ_max_ = 30.5°, θ_min_ = 3.6°
Absorption correction: multi-scan (*SADABS*; Sheldrick, 1997)	*h* = −20→20
*T*_min_ = 0.820, *T*_max_ = 0.909	*k* = −22→23
209835 measured reflections	*l* = −24→24

### Refinement

**Table:**

Refinement on *F*^2^	Primary atom site location: structure-invariant direct methods
Least-squares matrix: full	Secondary atom site location: difference Fourier map
*R*[*F*^2^ > 2σ(*F*^2^)] = 0.048	Hydrogen site location: inferred from neighbouring sites
*wR*(*F*^2^) = 0.149	H-atom parameters constrained
*S* = 1.06	*w* = 1/[σ^2^(*F*_o_^2^) + (0.0699*P*)^2^ + 12.1066*P*] where *P* = (*F*_o_^2^ + 2*F*_c_^2^)/3
22527 reflections	(Δ/σ)_max_ = 0.001
970 parameters	Δρ_max_ = 2.29 e Å^−^^3^
18 restraints	Δρ_min_ = −3.96 e Å^−^^3^

### Special details

**Table:**

Geometry. All e.s.d.'s (except the e.s.d. in the dihedral angle between two l.s. planes) are estimated using the full covariance matrix. The cell e.s.d.'s are taken into account individually in the estimation of e.s.d.'s in distances, angles and torsion angles; correlations between e.s.d.'s in cell parameters are only used when they are defined by crystal symmetry. An approximate (isotropic) treatment of cell e.s.d.'s is used for estimating e.s.d.'s involving l.s. planes.
Refinement. Refinement of *F*^2^ against ALL reflections. The weighted *R*-factor *wR* and goodness of fit *S* are based on *F*^2^, conventional *R*-factors *R* are based on *F*, with *F* set to zero for negative *F*^2^. The threshold expression of *F*^2^ > σ(*F*^2^) is used only for calculating *R*-factors(gt) *etc*. and is not relevant to the choice of reflections for refinement. *R*-factors based on *F*^2^ are statistically about twice as large as those based on *F*, and *R*- factors based on ALL data will be even larger.

### Fractional atomic coordinates and isotropic or equivalent isotropic displacement parameters (Å^2^)

**Table:**

	*x*	*y*	*z*	*U*_iso_*/*U*_eq_	Occ. (<1)
Mo1	0.99683 (3)	0.24876 (3)	0.75335 (2)	0.03534 (9)	
Mo2	0.61312 (2)	0.99765 (2)	0.42363 (2)	0.03034 (8)	
Mo3	0.40308 (2)	1.04781 (2)	0.39193 (2)	0.03038 (8)	
Mo4	0.40962 (2)	0.85933 (2)	0.45345 (2)	0.02699 (8)	
Mo5	0.62578 (3)	0.47005 (3)	1.09704 (2)	0.03444 (9)	
Mo6	0.60936 (3)	0.54152 (2)	0.91657 (2)	0.03324 (9)	
Mo7	0.44810 (3)	0.36044 (2)	0.94262 (2)	0.03202 (8)	
O1	0.5068 (2)	0.8872 (2)	0.39356 (17)	0.0319 (6)	
O2	0.5081 (2)	1.0402 (2)	0.35091 (17)	0.0347 (6)	
O3	0.3406 (2)	0.9289 (2)	0.37963 (17)	0.0323 (6)	
O4	0.5000	1.0000	0.5000	0.0235 (7)	
O5	0.6645 (2)	0.9530 (2)	0.51771 (18)	0.0333 (6)	
O6	0.6889 (3)	0.9911 (2)	0.3638 (2)	0.0444 (8)	
O7	0.6644 (2)	1.1105 (2)	0.47321 (18)	0.0322 (6)	
O8	0.3311 (3)	1.0869 (2)	0.3187 (2)	0.0473 (8)	
O9	0.5019 (2)	0.8446 (2)	0.54449 (18)	0.0328 (6)	
O10	0.3399 (2)	0.7580 (2)	0.41485 (19)	0.0358 (6)	
O11	0.6876 (2)	0.5073 (2)	1.0047 (2)	0.0387 (7)	
O12	0.5612 (2)	0.3641 (2)	1.0337 (2)	0.0395 (7)	
O13	0.5383 (2)	0.4161 (2)	0.8841 (2)	0.0383 (7)	
O14	0.5000	0.5000	1.0000	0.0268 (7)	
O15	0.7210 (3)	0.4530 (3)	1.1671 (2)	0.0492 (9)	
O16	0.5231 (2)	0.4508 (2)	1.14961 (19)	0.0397 (7)	
O17	0.6230 (2)	0.6448 (2)	0.9692 (2)	0.0381 (7)	
O18	0.6811 (3)	0.5688 (3)	0.8502 (2)	0.0496 (9)	
O19	0.3487 (2)	0.4064 (2)	0.88400 (19)	0.0368 (7)	
O20	0.4043 (3)	0.2565 (2)	0.9048 (2)	0.0444 (8)	
N1	0.9233 (2)	0.2980 (2)	0.65073 (19)	0.0254 (6)	
N2	1.0224 (2)	0.3776 (2)	0.79494 (18)	0.0238 (6)	
N3	0.8733 (2)	0.20271 (19)	0.80264 (17)	0.0210 (5)	
N4	1.0600 (2)	0.2127 (2)	0.86745 (19)	0.0235 (6)	
N5	0.9833 (2)	0.1286 (2)	0.6943 (2)	0.0267 (6)	
N6	1.1307 (2)	0.27394 (19)	0.71597 (18)	0.0217 (5)	
C1	0.8802 (3)	0.2555 (3)	0.5772 (2)	0.0319 (8)	
H1	0.8757	0.1965	0.5707	0.038*	
C2	0.8421 (3)	0.2944 (4)	0.5105 (3)	0.0404 (11)	
H2	0.8131	0.2623	0.4595	0.048*	
C3	0.8464 (3)	0.3794 (4)	0.5181 (3)	0.0435 (11)	
C4	0.8899 (3)	0.4232 (3)	0.5950 (3)	0.0401 (10)	
H4	0.8929	0.4816	0.6031	0.048*	
C5	0.9286 (3)	0.3813 (3)	0.6597 (2)	0.0283 (7)	
C6	0.9795 (3)	0.4249 (2)	0.7413 (2)	0.0277 (7)	
C7	0.9852 (4)	0.5087 (3)	0.7627 (3)	0.0417 (10)	
H7	0.9541	0.5405	0.7242	0.050*	
C8	1.0366 (4)	0.5466 (3)	0.8404 (3)	0.0448 (11)	
C9	1.0783 (4)	0.4972 (3)	0.8944 (3)	0.0392 (10)	
H9	1.1128	0.5202	0.9482	0.047*	
C10	1.0699 (3)	0.4140 (3)	0.8700 (2)	0.0303 (8)	
H10	1.0992	0.3810	0.9082	0.036*	
C11	0.8025 (5)	0.4227 (5)	0.4454 (4)	0.070 (2)	
C12	0.8343 (7)	0.4023 (7)	0.3717 (4)	0.099 (3)	
H12A	0.9078	0.4193	0.3841	0.149*	
H12B	0.8096	0.4342	0.3272	0.149*	
H12C	0.8062	0.3403	0.3560	0.149*	
C13	0.6889 (5)	0.3903 (7)	0.4301 (6)	0.108 (4)	
H13A	0.6656	0.3272	0.4229	0.162*	
H13B	0.6586	0.4136	0.3813	0.162*	
H13C	0.6693	0.4093	0.4764	0.162*	
C14	0.8397 (7)	0.5214 (6)	0.4618 (5)	0.101 (3)	
H14A	0.8149	0.5376	0.5062	0.152*	
H14B	0.8146	0.5476	0.4131	0.152*	
H14C	0.9134	0.5418	0.4766	0.152*	
C15	1.0444 (6)	0.6384 (4)	0.8650 (4)	0.0672 (19)	
C16	1.1517 (8)	0.6888 (5)	0.9009 (7)	0.122 (4)	
H16A	1.1738	0.6710	0.9550	0.183*	
H16B	1.1588	0.7504	0.9044	0.183*	
H16C	1.1932	0.6780	0.8667	0.183*	
C17	0.9779 (6)	0.6346 (4)	0.9231 (4)	0.070 (2)	
H17A	0.9075	0.6056	0.8949	0.106*	
H17B	0.9853	0.6932	0.9427	0.106*	
H17C	0.9981	0.6024	0.9689	0.106*	
C18	1.0087 (11)	0.6861 (5)	0.7917 (6)	0.127 (5)	
H18A	1.0446	0.6821	0.7506	0.191*	
H18B	1.0226	0.7469	0.8089	0.191*	
H18C	0.9363	0.6597	0.7690	0.191*	
C19	0.7789 (3)	0.1996 (3)	0.7651 (2)	0.0259 (7)	
H19	0.7669	0.2197	0.7127	0.031*	
C20	0.6987 (3)	0.1684 (3)	0.7992 (2)	0.0301 (8)	
H20	0.6330	0.1661	0.7699	0.036*	
C21	0.7144 (3)	0.1405 (3)	0.8764 (2)	0.0289 (7)	
C22	0.8135 (3)	0.1458 (2)	0.9165 (2)	0.0244 (7)	
H22	0.8279	0.1288	0.9700	0.029*	
C23	0.8901 (2)	0.1759 (2)	0.8780 (2)	0.0203 (6)	
C24	0.9968 (2)	0.1808 (2)	0.9155 (2)	0.0199 (6)	
C25	1.0304 (3)	0.1568 (2)	0.9913 (2)	0.0237 (6)	
H25	0.9844	0.1351	1.0235	0.028*	
C26	1.1327 (3)	0.1641 (2)	1.0216 (2)	0.0259 (7)	
C27	1.1951 (3)	0.1945 (3)	0.9713 (2)	0.0297 (8)	
H27	1.2643	0.1991	0.9885	0.036*	
C28	1.1570 (3)	0.2183 (3)	0.8961 (2)	0.0294 (8)	
H28	1.2017	0.2398	0.8628	0.035*	
C29	0.6257 (3)	0.1030 (3)	0.9135 (3)	0.0362 (9)	
C30	0.6580 (5)	0.0679 (5)	0.9960 (4)	0.0426 (17)	0.707 (9)
H30A	0.6887	0.0232	0.9892	0.064*	0.707 (9)
H30B	0.5990	0.0432	1.0172	0.064*	0.707 (9)
H30C	0.7068	0.1150	1.0340	0.064*	0.707 (9)
C31	0.5490 (5)	0.0297 (6)	0.8561 (5)	0.051 (2)	0.707 (9)
H31A	0.5190	0.0529	0.8071	0.077*	0.707 (9)
H31B	0.4963	−0.0007	0.8822	0.077*	0.707 (9)
H31C	0.5821	−0.0104	0.8418	0.077*	0.707 (9)
C32	0.5837 (6)	0.1759 (6)	0.9269 (5)	0.0494 (19)	0.707 (9)
H32A	0.6355	0.2215	0.9647	0.074*	0.707 (9)
H32B	0.5253	0.1535	0.9494	0.074*	0.707 (9)
H32C	0.5633	0.1995	0.8753	0.074*	0.707 (9)
C30'	0.6101 (14)	0.0063 (12)	0.9219 (15)	0.057 (6)	0.293 (9)
H30D	0.5505	−0.0188	0.9424	0.086*	0.293 (9)
H30E	0.6692	−0.0013	0.9597	0.086*	0.293 (9)
H30F	0.6006	−0.0227	0.8691	0.086*	0.293 (9)
C31'	0.5185 (13)	0.1027 (18)	0.8539 (13)	0.065 (7)	0.293 (9)
H31D	0.5101	0.0747	0.8009	0.098*	0.293 (9)
H31E	0.5176	0.1622	0.8479	0.098*	0.293 (9)
H31F	0.4634	0.0712	0.8772	0.098*	0.293 (9)
C32'	0.6384 (16)	0.1491 (15)	0.9917 (13)	0.061 (6)	0.293 (9)
H32D	0.5749	0.1309	1.0078	0.091*	0.293 (9)
H32E	0.6581	0.2113	0.9864	0.091*	0.293 (9)
H32F	0.6907	0.1355	1.0329	0.091*	0.293 (9)
C33	1.1731 (3)	0.1390 (3)	1.1056 (2)	0.0326 (8)	
C34	1.1487 (5)	0.1907 (4)	1.1679 (3)	0.0394 (14)	0.769 (8)
H34A	1.0757	0.1781	1.1570	0.059*	0.769 (8)
H34B	1.1802	0.2524	1.1647	0.059*	0.769 (8)
H34C	1.1745	0.1747	1.2221	0.059*	0.769 (8)
C35	1.1306 (5)	0.0446 (4)	1.1088 (4)	0.0413 (15)	0.769 (8)
H35A	1.0583	0.0297	1.1045	0.062*	0.769 (8)
H35B	1.1637	0.0274	1.1602	0.062*	0.769 (8)
H35C	1.1415	0.0144	1.0640	0.062*	0.769 (8)
C36	1.2911 (5)	0.1643 (5)	1.1269 (4)	0.0484 (18)	0.769 (8)
H36A	1.3160	0.1517	1.1826	0.073*	0.769 (8)
H36B	1.3195	0.2260	1.1214	0.073*	0.769 (8)
H36C	1.3112	0.1310	1.0899	0.073*	0.769 (8)
C34'	1.072 (2)	0.0766 (17)	1.1417 (13)	0.051 (6)	0.231 (8)
H34D	1.0981	0.0539	1.1925	0.077*	0.231 (8)
H34E	1.0304	0.0286	1.1017	0.077*	0.231 (8)
H34F	1.0318	0.1125	1.1512	0.077*	0.231 (8)
C35'	1.220 (2)	0.0699 (15)	1.0977 (13)	0.050 (7)	0.231 (8)
H35D	1.2573	0.0830	1.0560	0.076*	0.231 (8)
H35E	1.1670	0.0142	1.0824	0.076*	0.231 (8)
H35F	1.2658	0.0673	1.1494	0.076*	0.231 (8)
C36'	1.2255 (17)	0.2049 (12)	1.1630 (10)	0.038 (5)	0.231 (8)
H36D	1.2510	0.1818	1.2136	0.056*	0.231 (8)
H36E	1.1813	0.2375	1.1717	0.056*	0.231 (8)
H36F	1.2821	0.2431	1.1452	0.056*	0.231 (8)
C37	0.9049 (3)	0.0565 (3)	0.6843 (3)	0.0324 (8)	
H37	0.8451	0.0604	0.6961	0.039*	
C38	0.9063 (3)	−0.0231 (3)	0.6579 (3)	0.0330 (8)	
H38	0.8478	−0.0719	0.6503	0.040*	
C39	0.9934 (3)	−0.0316 (2)	0.6425 (2)	0.0279 (7)	
C40	1.0754 (3)	0.0438 (2)	0.6528 (2)	0.0272 (7)	
H40	1.1367	0.0412	0.6431	0.033*	
C41	1.0677 (3)	0.1224 (2)	0.6770 (2)	0.0233 (6)	
C42	1.1493 (3)	0.2056 (2)	0.6846 (2)	0.0217 (6)	
C43	1.2370 (3)	0.2160 (2)	0.6605 (2)	0.0262 (7)	
H43	1.2486	0.1673	0.6391	0.031*	
C44	1.3089 (3)	0.2975 (3)	0.6675 (2)	0.0259 (7)	
C45	1.2886 (3)	0.3659 (2)	0.7003 (2)	0.0256 (7)	
H45	1.3353	0.4223	0.7067	0.031*	
C46	1.1997 (3)	0.3517 (2)	0.7237 (2)	0.0239 (7)	
H46	1.1872	0.3993	0.7463	0.029*	
C47	1.0013 (4)	−0.1181 (3)	0.6144 (3)	0.0361 (9)	
C48	0.9069 (5)	−0.1927 (4)	0.6133 (5)	0.072 (2)	
H48A	0.8500	−0.1852	0.5725	0.108*	
H48B	0.8926	−0.1936	0.6665	0.108*	
H48C	0.9173	−0.2471	0.6000	0.108*	
C49	1.0902 (5)	−0.1373 (4)	0.6736 (5)	0.0699 (19)	
H49A	1.0912	−0.1951	0.6592	0.105*	
H49B	1.0823	−0.1344	0.7288	0.105*	
H49C	1.1534	−0.0946	0.6704	0.105*	
C50	1.0258 (9)	−0.1114 (4)	0.5349 (4)	0.106 (4)	
H50A	0.9729	−0.0967	0.4954	0.158*	
H50B	1.0306	−0.1667	0.5169	0.158*	
H50C	1.0902	−0.0663	0.5398	0.158*	
C51	1.4068 (3)	0.3083 (3)	0.6431 (3)	0.0315 (8)	
C52	1.4673 (3)	0.2620 (3)	0.7014 (3)	0.0355 (9)	
H52A	1.4844	0.2895	0.7562	0.053*	
H52B	1.5292	0.2654	0.6854	0.053*	
H52C	1.4274	0.2014	0.7002	0.053*	
C53	1.3823 (3)	0.2681 (3)	0.5561 (3)	0.0400 (10)	
H53A	1.4450	0.2748	0.5403	0.060*	
H53B	1.3416	0.2973	0.5194	0.060*	
H53C	1.3448	0.2067	0.5534	0.060*	
C54	1.4687 (4)	0.4031 (3)	0.6466 (4)	0.0453 (11)	
H54A	1.4867	0.4290	0.7021	0.068*	
H54B	1.4290	0.4337	0.6107	0.068*	
H54C	1.5300	0.4074	0.6294	0.068*	
N100	0.7462 (11)	0.5880 (7)	0.6388 (8)	0.153 (5)	
C100	0.6882 (10)	0.6173 (7)	0.6509 (7)	0.101 (3)	
C101	0.6161 (8)	0.6533 (7)	0.6666 (6)	0.101 (3)	
H10A	0.5500	0.6231	0.6310	0.152*	
H10B	0.6351	0.7145	0.6566	0.152*	
H10C	0.6133	0.6474	0.7230	0.152*	
N101	0.8348 (6)	0.3898 (5)	0.9056 (5)	0.098 (2)	
C102	0.7866 (5)	0.4003 (5)	0.8441 (5)	0.0668 (17)	
C103	0.7270 (5)	0.4129 (5)	0.7680 (5)	0.074 (2)	
H10D	0.6604	0.4112	0.7735	0.112*	
H10E	0.7199	0.3671	0.7277	0.112*	
H10F	0.7599	0.4690	0.7505	0.112*	
N102	0.3105 (8)	0.8518 (8)	0.7853 (8)	0.151 (4)	
C104	0.3658 (7)	0.8141 (6)	0.7863 (6)	0.092 (3)	
C105	0.4404 (8)	0.7694 (7)	0.7885 (8)	0.129 (4)	
H10G	0.4593	0.7730	0.7370	0.194*	
H10H	0.4119	0.7088	0.7977	0.194*	
H10I	0.5002	0.7966	0.8324	0.194*	
N103	0.1269 (7)	−0.0439 (5)	0.8867 (4)	0.098 (2)	
C106	0.1899 (6)	−0.0034 (4)	0.8614 (4)	0.0673 (18)	
C107	0.2727 (5)	0.0497 (4)	0.8297 (4)	0.0632 (16)	
H10J	0.3230	0.0895	0.8734	0.095*	
H10K	0.2464	0.0828	0.7870	0.095*	
H10L	0.3038	0.0124	0.8076	0.095*	

### Atomic displacement parameters (Å^2^)

**Table:**

	*U*^11^	*U*^22^	*U*^33^	*U*^12^	*U*^13^	*U*^23^
Mo1	0.03491 (18)	0.0378 (2)	0.03418 (19)	0.01294 (15)	0.00823 (15)	0.00203 (15)
Mo2	0.02350 (15)	0.03969 (19)	0.02892 (16)	0.00788 (13)	0.01123 (12)	−0.00434 (14)
Mo3	0.02459 (15)	0.03874 (19)	0.02395 (16)	0.01084 (13)	−0.00232 (12)	0.00086 (13)
Mo4	0.02027 (14)	0.03305 (17)	0.02492 (15)	0.00682 (12)	0.00255 (11)	−0.00479 (12)
Mo5	0.02564 (16)	0.0380 (2)	0.03497 (19)	0.00841 (14)	0.00081 (13)	−0.00321 (15)
Mo6	0.02785 (16)	0.03568 (19)	0.03500 (18)	0.00374 (14)	0.01365 (14)	−0.00431 (14)
Mo7	0.02650 (16)	0.02871 (17)	0.03661 (19)	0.00291 (13)	0.00745 (13)	−0.00894 (14)
O1	0.0261 (13)	0.0410 (16)	0.0277 (14)	0.0088 (12)	0.0077 (11)	−0.0054 (12)
O2	0.0353 (15)	0.0452 (17)	0.0224 (13)	0.0102 (13)	0.0078 (11)	−0.0004 (12)
O3	0.0235 (12)	0.0409 (16)	0.0268 (13)	0.0090 (11)	−0.0028 (10)	−0.0060 (12)
O4	0.0186 (15)	0.0321 (19)	0.0186 (16)	0.0076 (14)	0.0031 (12)	−0.0037 (14)
O5	0.0228 (12)	0.0414 (16)	0.0370 (15)	0.0153 (12)	0.0031 (11)	−0.0041 (13)
O6	0.0398 (17)	0.052 (2)	0.0465 (19)	0.0106 (15)	0.0245 (15)	−0.0067 (16)
O7	0.0224 (12)	0.0369 (15)	0.0358 (15)	0.0049 (11)	0.0101 (11)	−0.0029 (12)
O8	0.0429 (18)	0.053 (2)	0.0386 (18)	0.0158 (16)	−0.0041 (14)	0.0070 (15)
O9	0.0292 (14)	0.0378 (16)	0.0304 (14)	0.0117 (12)	0.0040 (11)	0.0006 (12)
O10	0.0273 (14)	0.0351 (16)	0.0403 (16)	0.0062 (12)	0.0042 (12)	−0.0050 (13)
O11	0.0240 (13)	0.0450 (18)	0.0469 (18)	0.0089 (12)	0.0109 (12)	−0.0043 (14)
O12	0.0353 (16)	0.0366 (16)	0.0467 (18)	0.0130 (13)	0.0083 (14)	−0.0016 (14)
O13	0.0338 (15)	0.0399 (17)	0.0388 (16)	0.0050 (13)	0.0133 (13)	−0.0103 (13)
O14	0.0210 (16)	0.0294 (19)	0.0272 (18)	0.0051 (14)	0.0043 (14)	−0.0081 (15)
O15	0.0374 (17)	0.056 (2)	0.048 (2)	0.0179 (16)	−0.0046 (15)	−0.0009 (17)
O16	0.0398 (16)	0.0462 (18)	0.0309 (15)	0.0098 (14)	0.0095 (13)	0.0009 (13)
O17	0.0333 (15)	0.0335 (16)	0.0433 (17)	0.0016 (12)	0.0134 (13)	−0.0031 (13)
O18	0.0465 (19)	0.053 (2)	0.051 (2)	0.0070 (16)	0.0275 (17)	0.0001 (17)
O19	0.0259 (13)	0.0387 (16)	0.0369 (16)	0.0034 (12)	−0.0001 (12)	−0.0105 (13)
O20	0.0376 (17)	0.0375 (17)	0.055 (2)	0.0061 (14)	0.0123 (15)	−0.0139 (15)
N1	0.0239 (14)	0.0329 (16)	0.0217 (14)	0.0089 (12)	0.0099 (11)	0.0015 (12)
N2	0.0241 (14)	0.0267 (15)	0.0227 (14)	0.0080 (12)	0.0095 (11)	−0.0003 (12)
N3	0.0213 (13)	0.0230 (14)	0.0183 (13)	0.0083 (11)	0.0027 (10)	−0.0032 (11)
N4	0.0177 (12)	0.0233 (14)	0.0264 (15)	0.0044 (11)	0.0021 (11)	0.0023 (12)
N5	0.0214 (13)	0.0250 (15)	0.0318 (16)	0.0060 (12)	0.0048 (12)	0.0003 (12)
N6	0.0234 (13)	0.0198 (13)	0.0213 (13)	0.0069 (11)	0.0045 (11)	0.0014 (11)
C1	0.0264 (17)	0.047 (2)	0.0200 (16)	0.0073 (16)	0.0065 (13)	−0.0013 (16)
C2	0.0266 (18)	0.067 (3)	0.0211 (18)	0.0058 (19)	0.0041 (14)	0.0053 (19)
C3	0.029 (2)	0.066 (3)	0.033 (2)	0.012 (2)	0.0067 (16)	0.020 (2)
C4	0.037 (2)	0.048 (3)	0.040 (2)	0.019 (2)	0.0098 (18)	0.021 (2)
C5	0.0274 (17)	0.034 (2)	0.0285 (18)	0.0127 (15)	0.0118 (14)	0.0082 (15)
C6	0.0281 (17)	0.0251 (17)	0.0330 (19)	0.0088 (14)	0.0128 (15)	0.0031 (15)
C7	0.057 (3)	0.030 (2)	0.048 (3)	0.019 (2)	0.024 (2)	0.0078 (19)
C8	0.060 (3)	0.025 (2)	0.056 (3)	0.0067 (19)	0.033 (2)	−0.0047 (19)
C9	0.041 (2)	0.034 (2)	0.038 (2)	0.0001 (18)	0.0160 (18)	−0.0110 (18)
C10	0.0303 (18)	0.0298 (19)	0.0277 (18)	0.0043 (15)	0.0078 (15)	−0.0072 (15)
C11	0.051 (3)	0.099 (5)	0.051 (3)	0.020 (3)	0.000 (3)	0.041 (3)
C12	0.097 (6)	0.156 (9)	0.037 (3)	0.029 (6)	0.014 (3)	0.046 (4)
C13	0.047 (4)	0.151 (9)	0.115 (7)	0.033 (5)	−0.005 (4)	0.078 (6)
C14	0.109 (7)	0.100 (6)	0.082 (5)	0.036 (5)	−0.007 (5)	0.060 (5)
C15	0.107 (5)	0.028 (2)	0.073 (4)	0.015 (3)	0.040 (4)	−0.006 (3)
C16	0.150 (9)	0.039 (4)	0.177 (10)	−0.017 (4)	0.100 (8)	−0.032 (5)
C17	0.126 (6)	0.042 (3)	0.070 (4)	0.044 (4)	0.052 (4)	0.006 (3)
C18	0.282 (14)	0.047 (4)	0.106 (7)	0.079 (6)	0.109 (8)	0.030 (4)
C19	0.0214 (15)	0.0330 (19)	0.0221 (16)	0.0089 (14)	0.0027 (12)	−0.0023 (14)
C20	0.0216 (16)	0.038 (2)	0.0314 (19)	0.0118 (15)	0.0054 (14)	−0.0007 (16)
C21	0.0249 (16)	0.0317 (19)	0.0326 (19)	0.0091 (14)	0.0118 (14)	−0.0029 (15)
C22	0.0260 (16)	0.0257 (17)	0.0244 (16)	0.0098 (13)	0.0096 (13)	0.0012 (13)
C23	0.0212 (14)	0.0189 (15)	0.0204 (15)	0.0071 (12)	0.0038 (12)	−0.0026 (12)
C24	0.0212 (14)	0.0168 (14)	0.0220 (15)	0.0062 (12)	0.0055 (12)	−0.0009 (12)
C25	0.0294 (17)	0.0214 (16)	0.0218 (16)	0.0096 (13)	0.0070 (13)	0.0010 (13)
C26	0.0338 (18)	0.0197 (16)	0.0226 (16)	0.0118 (14)	−0.0003 (13)	−0.0001 (13)
C27	0.0215 (16)	0.0288 (18)	0.0325 (19)	0.0062 (14)	−0.0030 (14)	0.0047 (15)
C28	0.0201 (15)	0.033 (2)	0.0314 (19)	0.0068 (14)	0.0013 (13)	0.0078 (15)
C29	0.0258 (18)	0.050 (3)	0.039 (2)	0.0131 (17)	0.0161 (16)	0.0061 (19)
C30	0.027 (3)	0.064 (5)	0.039 (3)	0.011 (3)	0.016 (2)	0.016 (3)
C31	0.025 (3)	0.066 (5)	0.052 (4)	−0.001 (3)	0.012 (3)	−0.005 (4)
C32	0.042 (4)	0.068 (5)	0.055 (4)	0.034 (4)	0.022 (3)	0.008 (4)
C30'	0.039 (9)	0.043 (10)	0.095 (16)	0.008 (7)	0.033 (10)	0.022 (10)
C31'	0.033 (8)	0.12 (2)	0.056 (12)	0.027 (11)	0.022 (8)	0.023 (12)
C32'	0.055 (11)	0.072 (14)	0.071 (13)	0.021 (10)	0.044 (10)	0.004 (11)
C33	0.039 (2)	0.037 (2)	0.0243 (18)	0.0197 (17)	0.0015 (15)	0.0054 (16)
C34	0.050 (4)	0.045 (3)	0.024 (3)	0.020 (3)	0.002 (2)	0.001 (2)
C35	0.060 (4)	0.031 (3)	0.035 (3)	0.018 (3)	0.009 (3)	0.011 (2)
C36	0.036 (3)	0.074 (5)	0.036 (3)	0.022 (3)	0.003 (2)	0.023 (3)
C34'	0.067 (15)	0.057 (14)	0.030 (10)	0.024 (12)	0.007 (9)	0.021 (10)
C35'	0.087 (19)	0.041 (12)	0.033 (10)	0.044 (13)	0.001 (10)	0.008 (8)
C36'	0.056 (13)	0.032 (9)	0.018 (7)	0.018 (9)	−0.009 (7)	−0.008 (6)
C37	0.0238 (17)	0.0276 (19)	0.044 (2)	0.0052 (14)	0.0083 (16)	−0.0027 (17)
C38	0.0284 (18)	0.0238 (18)	0.043 (2)	0.0041 (14)	0.0069 (16)	−0.0012 (16)
C39	0.0347 (19)	0.0243 (17)	0.0231 (17)	0.0083 (15)	0.0056 (14)	−0.0022 (14)
C40	0.0311 (18)	0.0268 (18)	0.0250 (17)	0.0088 (14)	0.0095 (14)	0.0000 (14)
C41	0.0237 (15)	0.0247 (16)	0.0219 (16)	0.0076 (13)	0.0061 (12)	0.0007 (13)
C42	0.0226 (15)	0.0233 (16)	0.0194 (15)	0.0082 (13)	0.0043 (12)	0.0014 (12)
C43	0.0254 (16)	0.0261 (17)	0.0284 (18)	0.0091 (14)	0.0078 (14)	0.0001 (14)
C44	0.0247 (16)	0.0297 (18)	0.0246 (17)	0.0094 (14)	0.0076 (13)	0.0047 (14)
C45	0.0239 (16)	0.0219 (16)	0.0296 (18)	0.0045 (13)	0.0077 (13)	0.0035 (14)
C46	0.0231 (15)	0.0240 (16)	0.0247 (16)	0.0078 (13)	0.0053 (13)	0.0008 (13)
C47	0.048 (2)	0.0223 (18)	0.039 (2)	0.0076 (17)	0.0171 (19)	−0.0019 (16)
C48	0.061 (4)	0.039 (3)	0.107 (6)	0.006 (3)	0.018 (4)	−0.023 (3)
C49	0.076 (4)	0.039 (3)	0.096 (5)	0.028 (3)	0.010 (4)	0.005 (3)
C50	0.252 (12)	0.045 (3)	0.057 (4)	0.061 (5)	0.090 (6)	0.010 (3)
C51	0.0276 (17)	0.034 (2)	0.033 (2)	0.0085 (15)	0.0111 (15)	0.0019 (16)
C52	0.0311 (19)	0.044 (2)	0.036 (2)	0.0175 (18)	0.0098 (16)	−0.0013 (18)
C53	0.037 (2)	0.054 (3)	0.037 (2)	0.018 (2)	0.0172 (18)	0.006 (2)
C54	0.034 (2)	0.038 (2)	0.069 (3)	0.0072 (18)	0.027 (2)	0.008 (2)
N100	0.188 (12)	0.114 (8)	0.193 (12)	0.050 (8)	0.111 (10)	0.065 (8)
C100	0.122 (9)	0.082 (7)	0.094 (7)	0.018 (6)	0.034 (6)	0.035 (5)
C101	0.100 (7)	0.103 (7)	0.079 (6)	0.016 (6)	0.003 (5)	0.016 (5)
N101	0.083 (5)	0.105 (6)	0.091 (5)	0.016 (4)	0.013 (4)	−0.012 (4)
C102	0.049 (3)	0.068 (4)	0.077 (5)	0.013 (3)	0.012 (3)	−0.018 (4)
C103	0.050 (3)	0.082 (5)	0.089 (5)	0.027 (3)	0.005 (3)	−0.016 (4)
N102	0.115 (8)	0.156 (10)	0.206 (12)	0.065 (8)	0.053 (8)	0.061 (9)
C104	0.067 (5)	0.080 (6)	0.116 (7)	0.012 (4)	0.010 (5)	0.023 (5)
C105	0.090 (7)	0.098 (8)	0.187 (13)	0.036 (6)	0.004 (8)	−0.016 (8)
N103	0.137 (7)	0.090 (5)	0.068 (4)	0.016 (5)	0.053 (4)	0.012 (4)
C106	0.106 (6)	0.057 (4)	0.042 (3)	0.020 (4)	0.031 (3)	0.008 (3)
C107	0.067 (4)	0.059 (4)	0.053 (3)	0.013 (3)	0.005 (3)	0.012 (3)

### Geometric parameters (Å, °)

**Table:**

Mo1—N1	2.117 (3)	C25—C26	1.409 (5)
Mo1—N2	2.113 (3)	C25—H25	0.9500
Mo1—N3	2.090 (3)	C26—C27	1.379 (6)
Mo1—N4	2.113 (3)	C26—C33	1.524 (5)
Mo1—N5	2.138 (3)	C27—C28	1.380 (5)
Mo1—N6	2.103 (3)	C27—H27	0.9500
Mo2—O1	1.953 (3)	C28—H28	0.9500
Mo2—O2	2.015 (3)	C29—C32'	1.48 (2)
Mo2—O4	2.3300 (3)	C29—C31	1.515 (9)
Mo2—O5	1.853 (3)	C29—C32	1.528 (8)
Mo2—O6	1.691 (3)	C29—C30'	1.538 (19)
Mo2—O7	1.878 (3)	C29—C30	1.545 (8)
Mo3—O8	1.683 (3)	C29—C31'	1.634 (19)
Mo3—O2	1.850 (3)	C30—H30A	0.9800
Mo3—O3	1.864 (3)	C30—H30B	0.9800
Mo3—O9^i^	1.992 (3)	C30—H30C	0.9800
Mo3—O5^i^	2.014 (3)	C31—H31A	0.9800
Mo3—O4	2.3256 (3)	C31—H31B	0.9800
Mo4—O10	1.690 (3)	C31—H31C	0.9800
Mo4—O9	1.859 (3)	C32—H32A	0.9800
Mo4—O1	1.878 (3)	C32—H32B	0.9800
Mo4—O7^i^	1.978 (3)	C32—H32C	0.9800
Mo4—O3	1.990 (3)	C30'—H30D	0.9800
Mo4—O4	2.3007 (4)	C30'—H30E	0.9800
Mo5—O15	1.686 (3)	C30'—H30F	0.9800
Mo5—O16	1.860 (3)	C31'—H31D	0.9800
Mo5—O12	1.888 (3)	C31'—H31E	0.9800
Mo5—O19^ii^	1.949 (3)	C31'—H31F	0.9800
Mo5—O11	2.003 (3)	C32'—H32D	0.9800
Mo5—O14	2.3199 (4)	C32'—H32E	0.9800
Mo6—O18	1.687 (3)	C32'—H32F	0.9800
Mo6—O17	1.845 (3)	C33—C36'	1.357 (18)
Mo6—O11	1.851 (3)	C33—C35	1.483 (7)
Mo6—O13	2.001 (3)	C33—C35'	1.50 (2)
Mo6—O16^ii^	2.014 (3)	C33—C34	1.526 (7)
Mo6—O14	2.3289 (4)	C33—C36	1.573 (7)
Mo7—O20	1.688 (3)	C33—C34'	1.75 (3)
Mo7—O13	1.854 (3)	C34—H34A	0.9800
Mo7—O19	1.893 (3)	C34—H34B	0.9800
Mo7—O12	1.953 (3)	C34—H34C	0.9800
Mo7—O17^ii^	2.005 (3)	C35—H35A	0.9800
Mo7—O14	2.3067 (4)	C35—H35B	0.9800
O4—Mo4^i^	2.3007 (4)	C35—H35C	0.9800
O4—Mo3^i^	2.3256 (3)	C36—H36A	0.9800
O4—Mo2^i^	2.3300 (3)	C36—H36B	0.9800
O5—Mo3^i^	2.014 (3)	C36—H36C	0.9800
O7—Mo4^i^	1.978 (3)	C34'—H34D	0.9800
O9—Mo3^i^	1.992 (3)	C34'—H34E	0.9800
O14—Mo7^ii^	2.3067 (4)	C34'—H34F	0.9800
O14—Mo5^ii^	2.3199 (4)	C35'—H35D	0.9800
O14—Mo6^ii^	2.3289 (4)	C35'—H35E	0.9800
O16—Mo6^ii^	2.014 (3)	C35'—H35F	0.9800
O17—Mo7^ii^	2.005 (3)	C36'—H36D	0.9800
O19—Mo5^ii^	1.949 (3)	C36'—H36E	0.9800
N1—C5	1.345 (5)	C36'—H36F	0.9800
N1—C1	1.346 (5)	C37—C38	1.383 (6)
N2—C10	1.343 (5)	C37—H37	0.9500
N2—C6	1.356 (5)	C38—C39	1.390 (6)
N3—C19	1.344 (4)	C38—H38	0.9500
N3—C23	1.352 (4)	C39—C40	1.403 (5)
N4—C28	1.341 (4)	C39—C47	1.534 (5)
N4—C24	1.362 (4)	C40—C41	1.392 (5)
N5—C37	1.339 (5)	C40—H40	0.9500
N5—C41	1.352 (5)	C41—C42	1.486 (5)
N6—C46	1.339 (5)	C42—C43	1.385 (5)
N6—C42	1.359 (4)	C43—C44	1.403 (5)
C1—C2	1.386 (6)	C43—H43	0.9500
C1—H1	0.9500	C44—C45	1.385 (5)
C2—C3	1.373 (8)	C44—C51	1.527 (5)
C2—H2	0.9500	C45—C46	1.388 (5)
C3—C4	1.402 (7)	C45—H45	0.9500
C3—C11	1.532 (7)	C46—H46	0.9500
C4—C5	1.393 (5)	C47—C50	1.482 (7)
C4—H4	0.9500	C47—C48	1.524 (8)
C5—C6	1.478 (6)	C47—C49	1.546 (8)
C6—C7	1.388 (6)	C48—H48A	0.9800
C7—C8	1.396 (7)	C48—H48B	0.9800
C7—H7	0.9500	C48—H48C	0.9800
C8—C9	1.376 (8)	C49—H49A	0.9800
C8—C15	1.520 (7)	C49—H49B	0.9800
C9—C10	1.379 (6)	C49—H49C	0.9800
C9—H9	0.9500	C50—H50A	0.9800
C10—H10	0.9500	C50—H50B	0.9800
C11—C12	1.509 (11)	C50—H50C	0.9800
C11—C13	1.518 (9)	C51—C52	1.521 (6)
C11—C14	1.537 (12)	C51—C54	1.528 (6)
C12—H12A	0.9800	C51—C53	1.540 (6)
C12—H12B	0.9800	C52—H52A	0.9800
C12—H12C	0.9800	C52—H52B	0.9800
C13—H13A	0.9800	C52—H52C	0.9800
C13—H13B	0.9800	C53—H53A	0.9800
C13—H13C	0.9800	C53—H53B	0.9800
C14—H14A	0.9800	C53—H53C	0.9800
C14—H14B	0.9800	C54—H54A	0.9800
C14—H14C	0.9800	C54—H54B	0.9800
C15—C16	1.491 (12)	C54—H54C	0.9800
C15—C17	1.527 (9)	N100—C100	1.140 (15)
C15—C18	1.543 (11)	C100—C101	1.413 (16)
C16—H16A	0.9800	C101—H10A	0.9800
C16—H16B	0.9800	C101—H10B	0.9800
C16—H16C	0.9800	C101—H10C	0.9800
C17—H17A	0.9800	N101—C102	1.160 (10)
C17—H17B	0.9800	C102—C103	1.431 (10)
C17—H17C	0.9800	C103—H10D	0.9800
C18—H18A	0.9800	C103—H10E	0.9800
C18—H18B	0.9800	C103—H10F	0.9800
C18—H18C	0.9800	N102—C104	1.143 (13)
C19—C20	1.381 (5)	C104—C105	1.464 (14)
C19—H19	0.9500	C105—H10G	0.9800
C20—C21	1.387 (6)	C105—H10H	0.9800
C20—H20	0.9500	C105—H10I	0.9800
C21—C22	1.406 (5)	N103—C106	1.131 (10)
C21—C29	1.529 (5)	C106—C107	1.469 (9)
C22—C23	1.385 (5)	C107—H10J	0.9800
C22—H22	0.9500	C107—H10K	0.9800
C23—C24	1.497 (5)	C107—H10L	0.9800
C24—C25	1.373 (5)		
			
N1—Mo1—N2	77.40 (12)	H17A—C17—H17B	109.5
N3—Mo1—N4	76.77 (11)	C15—C17—H17C	109.5
N5—Mo1—N6	76.32 (12)	H17A—C17—H17C	109.5
N1—Mo1—N5	96.61 (13)	H17B—C17—H17C	109.5
N2—Mo1—N4	94.64 (12)	C15—C18—H18A	109.5
N3—Mo1—N1	97.09 (11)	C15—C18—H18B	109.5
N3—Mo1—N2	92.57 (11)	H18A—C18—H18B	109.5
N3—Mo1—N5	96.63 (12)	C15—C18—H18C	109.5
N4—Mo1—N5	92.18 (12)	H18A—C18—H18C	109.5
N6—Mo1—N1	93.56 (11)	H18B—C18—H18C	109.5
N6—Mo1—N2	95.42 (12)	N3—C19—C20	123.0 (3)
N6—Mo1—N4	93.45 (11)	N3—C19—H19	118.5
N1—Mo1—N4	169.85 (12)	C20—C19—H19	118.5
N2—Mo1—N5	169.60 (12)	C19—C20—C21	119.8 (3)
N3—Mo1—N6	167.87 (11)	C19—C20—H20	120.1
O6—Mo2—O5	103.56 (16)	C21—C20—H20	120.1
O6—Mo2—O7	106.04 (15)	C20—C21—C22	117.3 (3)
O5—Mo2—O7	91.98 (14)	C20—C21—C29	120.2 (4)
O6—Mo2—O1	101.47 (15)	C22—C21—C29	122.5 (4)
O5—Mo2—O1	88.35 (13)	C23—C22—C21	119.9 (3)
O7—Mo2—O1	151.59 (12)	C23—C22—H22	120.1
O6—Mo2—O2	103.23 (16)	C21—C22—H22	120.1
O5—Mo2—O2	152.79 (12)	N3—C23—C22	122.0 (3)
O7—Mo2—O2	85.14 (13)	N3—C23—C24	114.9 (3)
O1—Mo2—O2	81.79 (13)	C22—C23—C24	123.1 (3)
O6—Mo2—O4	176.21 (12)	N4—C24—C25	121.9 (3)
O5—Mo2—O4	77.99 (9)	N4—C24—C23	113.6 (3)
O7—Mo2—O4	77.26 (8)	C25—C24—C23	124.5 (3)
O1—Mo2—O4	75.03 (8)	C24—C25—C26	120.4 (3)
O2—Mo2—O4	74.98 (8)	C24—C25—H25	119.8
O8—Mo3—O2	105.27 (17)	C26—C25—H25	119.8
O8—Mo3—O3	104.80 (16)	C27—C26—C25	116.9 (3)
O2—Mo3—O3	93.33 (14)	C27—C26—C33	121.0 (3)
O8—Mo3—O9^i^	101.95 (16)	C25—C26—C33	122.0 (4)
O2—Mo3—O9^i^	87.17 (13)	C26—C27—C28	119.9 (3)
O3—Mo3—O9^i^	152.09 (12)	C26—C27—H27	120.0
O8—Mo3—O5^i^	101.24 (16)	C28—C27—H27	120.0
O2—Mo3—O5^i^	152.83 (12)	N4—C28—C27	123.3 (4)
O3—Mo3—O5^i^	85.49 (13)	N4—C28—H28	118.3
O9^i^—Mo3—O5^i^	81.62 (12)	C27—C28—H28	118.3
O8—Mo3—O4	175.76 (14)	C32'—C29—C31	139.3 (9)
O2—Mo3—O4	78.10 (9)	C32'—C29—C32	53.6 (10)
O3—Mo3—O4	77.34 (8)	C31—C29—C32	112.3 (5)
O9^i^—Mo3—O4	75.47 (8)	C32'—C29—C21	112.5 (8)
O5^i^—Mo3—O4	75.16 (8)	C31—C29—C21	108.2 (4)
O10—Mo4—O9	104.50 (15)	C32—C29—C21	107.5 (4)
O10—Mo4—O1	102.31 (14)	C32'—C29—C30'	111.3 (14)
O9—Mo4—O1	92.00 (13)	C31—C29—C30'	54.0 (10)
O10—Mo4—O7^i^	104.11 (14)	C32—C29—C30'	143.3 (8)
O9—Mo4—O7^i^	87.83 (13)	C21—C29—C30'	109.2 (7)
O1—Mo4—O7^i^	152.74 (13)	C32'—C29—C30	56.6 (10)
O10—Mo4—O3	101.32 (14)	C31—C29—C30	108.8 (5)
O9—Mo4—O3	153.91 (13)	C32—C29—C30	108.2 (5)
O1—Mo4—O3	86.04 (13)	C21—C29—C30	111.9 (4)
O7^i^—Mo4—O3	82.35 (13)	C30'—C29—C30	58.2 (10)
O10—Mo4—O4	176.91 (11)	C32'—C29—C31'	108.3 (12)
O9—Mo4—O4	78.57 (10)	C31—C29—C31'	52.1 (10)
O1—Mo4—O4	77.11 (9)	C32—C29—C31'	61.5 (10)
O7^i^—Mo4—O4	76.15 (9)	C21—C29—C31'	112.8 (7)
O3—Mo4—O4	75.64 (8)	C30'—C29—C31'	102.3 (13)
O15—Mo5—O16	103.44 (17)	C30—C29—C31'	135.1 (8)
O15—Mo5—O12	105.14 (17)	C29—C30—H30A	109.5
O16—Mo5—O12	91.53 (15)	C29—C30—H30B	109.5
O15—Mo5—O19^ii^	102.11 (16)	C29—C30—H30C	109.5
O16—Mo5—O19^ii^	88.74 (14)	C29—C31—H31A	109.5
O12—Mo5—O19^ii^	151.89 (14)	C29—C31—H31B	109.5
O15—Mo5—O11	102.95 (17)	C29—C31—H31C	109.5
O16—Mo5—O11	153.39 (14)	C29—C32—H32A	109.5
O12—Mo5—O11	84.85 (14)	C29—C32—H32B	109.5
O19^ii^—Mo5—O11	82.48 (14)	C29—C32—H32C	109.5
O15—Mo5—O14	177.23 (14)	C29—C30'—H30D	109.5
O16—Mo5—O14	78.19 (10)	C29—C30'—H30E	109.5
O12—Mo5—O14	76.95 (10)	H30D—C30'—H30E	109.5
O19^ii^—Mo5—O14	75.60 (9)	C29—C30'—H30F	109.5
O11—Mo5—O14	75.31 (9)	H30D—C30'—H30F	109.5
O18—Mo6—O17	104.67 (17)	H30E—C30'—H30F	109.5
O18—Mo6—O11	105.66 (17)	C29—C31'—H31D	109.5
O17—Mo6—O11	93.68 (15)	C29—C31'—H31E	109.5
O18—Mo6—O13	102.15 (16)	H31D—C31'—H31E	109.5
O17—Mo6—O13	151.92 (13)	C29—C31'—H31F	109.5
O11—Mo6—O13	86.92 (15)	H31D—C31'—H31F	109.5
O18—Mo6—O16^ii^	101.09 (17)	H31E—C31'—H31F	109.5
O17—Mo6—O16^ii^	86.14 (14)	C29—C32'—H32D	109.5
O11—Mo6—O16^ii^	152.35 (14)	C29—C32'—H32E	109.5
O13—Mo6—O16^ii^	80.70 (14)	H32D—C32'—H32E	109.5
O18—Mo6—O14	175.72 (14)	C29—C32'—H32F	109.5
O17—Mo6—O14	77.29 (10)	H32D—C32'—H32F	109.5
O11—Mo6—O14	77.86 (9)	H32E—C32'—H32F	109.5
O13—Mo6—O14	75.41 (9)	C36'—C33—C35	133.3 (9)
O16^ii^—Mo6—O14	75.14 (9)	C36'—C33—C35'	117.2 (13)
O20—Mo7—O13	105.36 (16)	C35—C33—C35'	51.3 (12)
O20—Mo7—O19	102.41 (16)	C36'—C33—C26	116.2 (8)
O13—Mo7—O19	91.73 (15)	C35—C33—C26	109.6 (4)
O20—Mo7—O12	104.03 (16)	C35'—C33—C26	108.7 (8)
O13—Mo7—O12	89.25 (15)	C35—C33—C34	112.5 (5)
O19—Mo7—O12	152.27 (13)	C35'—C33—C34	142.2 (9)
O20—Mo7—O17^ii^	101.04 (15)	C26—C33—C34	109.0 (4)
O13—Mo7—O17^ii^	153.51 (13)	C36'—C33—C36	62.9 (10)
O19—Mo7—O17^ii^	84.65 (14)	C35—C33—C36	109.1 (5)
O12—Mo7—O17^ii^	82.26 (14)	C35'—C33—C36	61.6 (12)
O20—Mo7—O14	175.94 (12)	C26—C33—C36	110.7 (4)
O13—Mo7—O14	78.68 (10)	C34—C33—C36	105.9 (5)
O19—Mo7—O14	76.94 (9)	C36'—C33—C34'	105.9 (14)
O12—Mo7—O14	76.09 (10)	C35—C33—C34'	47.9 (9)
O17^ii^—Mo7—O14	74.93 (9)	C35'—C33—C34'	97.7 (15)
Mo4—O1—Mo2	117.22 (15)	C26—C33—C34'	109.1 (8)
Mo3—O2—Mo2	116.84 (14)	C34—C33—C34'	68.0 (9)
Mo3—O3—Mo4	116.30 (13)	C36—C33—C34'	139.3 (8)
Mo4^i^—O4—Mo4	180.0	C33—C34—H34A	109.5
Mo4^i^—O4—Mo3	89.884 (13)	C33—C34—H34B	109.5
Mo4—O4—Mo3	90.116 (13)	C33—C34—H34C	109.5
Mo4^i^—O4—Mo3^i^	90.116 (13)	C33—C35—H35A	109.5
Mo4—O4—Mo3^i^	89.884 (13)	C33—C35—H35B	109.5
Mo3—O4—Mo3^i^	180.000 (1)	C33—C35—H35C	109.5
Mo4^i^—O4—Mo2	90.137 (13)	C33—C36—H36A	109.5
Mo4—O4—Mo2	89.863 (12)	C33—C36—H36B	109.5
Mo3—O4—Mo2	90.059 (13)	C33—C36—H36C	109.5
Mo3^i^—O4—Mo2	89.941 (13)	C33—C34'—H34D	109.5
Mo4^i^—O4—Mo2^i^	89.863 (13)	C33—C34'—H34E	109.5
Mo4—O4—Mo2^i^	90.137 (13)	C33—C34'—H34F	109.5
Mo3—O4—Mo2^i^	89.941 (13)	C33—C35'—H35D	109.5
Mo3^i^—O4—Mo2^i^	90.059 (13)	C33—C35'—H35E	109.5
Mo2—O4—Mo2^i^	180.000 (1)	C33—C35'—H35F	109.5
Mo2—O5—Mo3^i^	116.55 (13)	C33—C36'—H36D	109.5
Mo2—O7—Mo4^i^	116.44 (14)	C33—C36'—H36E	109.5
Mo4—O9—Mo3^i^	116.05 (16)	C33—C36'—H36F	109.5
Mo6—O11—Mo5	116.84 (15)	N5—C37—C38	123.5 (4)
Mo5—O12—Mo7	116.89 (16)	N5—C37—H37	118.2
Mo7—O13—Mo6	116.06 (15)	C38—C37—H37	118.2
Mo7—O14—Mo7^ii^	180.000 (1)	C37—C38—C39	119.8 (4)
Mo7—O14—Mo5^ii^	89.931 (14)	C37—C38—H38	120.1
Mo7^ii^—O14—Mo5^ii^	90.069 (14)	C39—C38—H38	120.1
Mo7—O14—Mo5	90.069 (14)	C38—C39—C40	116.7 (4)
Mo7^ii^—O14—Mo5	89.931 (14)	C38—C39—C47	122.8 (4)
Mo5^ii^—O14—Mo5	180.0	C40—C39—C47	120.5 (4)
Mo7—O14—Mo6^ii^	90.238 (13)	C41—C40—C39	120.5 (4)
Mo7^ii^—O14—Mo6^ii^	89.762 (13)	C41—C40—H40	119.8
Mo5^ii^—O14—Mo6^ii^	89.912 (15)	C39—C40—H40	119.8
Mo5—O14—Mo6^ii^	90.088 (15)	N5—C41—C40	121.7 (3)
Mo7—O14—Mo6	89.762 (13)	N5—C41—C42	114.8 (3)
Mo7^ii^—O14—Mo6	90.238 (13)	C40—C41—C42	123.5 (3)
Mo5^ii^—O14—Mo6	90.088 (15)	N6—C42—C43	121.0 (3)
Mo5—O14—Mo6	89.912 (15)	N6—C42—C41	114.5 (3)
Mo6^ii^—O14—Mo6	180.0	C43—C42—C41	124.4 (3)
Mo5—O16—Mo6^ii^	116.20 (16)	C42—C43—C44	120.8 (3)
Mo6—O17—Mo7^ii^	117.07 (16)	C42—C43—H43	119.6
Mo7—O19—Mo5^ii^	116.61 (14)	C44—C43—H43	119.6
C5—N1—C1	118.4 (4)	C45—C44—C43	117.1 (3)
C5—N1—Mo1	115.7 (3)	C45—C44—C51	122.3 (3)
C1—N1—Mo1	125.6 (3)	C43—C44—C51	120.6 (3)
C10—N2—C6	117.9 (3)	C44—C45—C46	119.7 (3)
C10—N2—Mo1	126.3 (3)	C44—C45—H45	120.2
C6—N2—Mo1	115.6 (2)	C46—C45—H45	120.2
C19—N3—C23	118.0 (3)	N6—C46—C45	123.0 (3)
C19—N3—Mo1	124.4 (2)	N6—C46—H46	118.5
C23—N3—Mo1	117.6 (2)	C45—C46—H46	118.5
C28—N4—C24	117.5 (3)	C50—C47—C48	112.4 (6)
C28—N4—Mo1	125.3 (3)	C50—C47—C39	109.5 (4)
C24—N4—Mo1	117.2 (2)	C48—C47—C39	112.0 (4)
C37—N5—C41	117.7 (3)	C50—C47—C49	106.1 (6)
C37—N5—Mo1	125.8 (3)	C48—C47—C49	107.3 (5)
C41—N5—Mo1	115.6 (2)	C39—C47—C49	109.2 (4)
C46—N6—C42	118.4 (3)	C47—C48—H48A	109.5
C46—N6—Mo1	124.3 (2)	C47—C48—H48B	109.5
C42—N6—Mo1	117.2 (2)	H48A—C48—H48B	109.5
N1—C1—C2	122.7 (4)	C47—C48—H48C	109.5
N1—C1—H1	118.7	H48A—C48—H48C	109.5
C2—C1—H1	118.7	H48B—C48—H48C	109.5
C3—C2—C1	120.1 (4)	C47—C49—H49A	109.5
C3—C2—H2	120.0	C47—C49—H49B	109.5
C1—C2—H2	120.0	H49A—C49—H49B	109.5
C2—C3—C4	117.2 (4)	C47—C49—H49C	109.5
C2—C3—C11	120.7 (5)	H49A—C49—H49C	109.5
C4—C3—C11	122.1 (6)	H49B—C49—H49C	109.5
C5—C4—C3	120.3 (5)	C47—C50—H50A	109.5
C5—C4—H4	119.9	C47—C50—H50B	109.5
C3—C4—H4	119.9	H50A—C50—H50B	109.5
N1—C5—C4	121.3 (4)	C47—C50—H50C	109.5
N1—C5—C6	115.7 (3)	H50A—C50—H50C	109.5
C4—C5—C6	122.9 (4)	H50B—C50—H50C	109.5
N2—C6—C7	121.4 (4)	C52—C51—C44	107.7 (3)
N2—C6—C5	115.3 (3)	C52—C51—C54	109.1 (4)
C7—C6—C5	123.3 (4)	C44—C51—C54	111.9 (3)
C6—C7—C8	120.2 (4)	C52—C51—C53	110.2 (4)
C6—C7—H7	119.9	C44—C51—C53	109.3 (3)
C8—C7—H7	119.9	C54—C51—C53	108.6 (4)
C9—C8—C7	117.6 (4)	C51—C52—H52A	109.5
C9—C8—C15	121.3 (5)	C51—C52—H52B	109.5
C7—C8—C15	121.1 (5)	H52A—C52—H52B	109.5
C8—C9—C10	119.8 (4)	C51—C52—H52C	109.5
C8—C9—H9	120.1	H52A—C52—H52C	109.5
C10—C9—H9	120.1	H52B—C52—H52C	109.5
N2—C10—C9	123.1 (4)	C51—C53—H53A	109.5
N2—C10—H10	118.4	C51—C53—H53B	109.5
C9—C10—H10	118.4	H53A—C53—H53B	109.5
C12—C11—C13	110.6 (7)	C51—C53—H53C	109.5
C12—C11—C3	111.0 (6)	H53A—C53—H53C	109.5
C13—C11—C3	107.7 (5)	H53B—C53—H53C	109.5
C12—C11—C14	107.0 (7)	C51—C54—H54A	109.5
C13—C11—C14	109.4 (7)	C51—C54—H54B	109.5
C3—C11—C14	111.1 (5)	H54A—C54—H54B	109.5
C11—C12—H12A	109.5	C51—C54—H54C	109.5
C11—C12—H12B	109.5	H54A—C54—H54C	109.5
H12A—C12—H12B	109.5	H54B—C54—H54C	109.5
C11—C12—H12C	109.5	N100—C100—C101	179.5 (12)
H12A—C12—H12C	109.5	C100—C101—H10A	109.5
H12B—C12—H12C	109.5	C100—C101—H10B	109.5
C11—C13—H13A	109.5	H10A—C101—H10B	109.5
C11—C13—H13B	109.5	C100—C101—H10C	109.5
H13A—C13—H13B	109.5	H10A—C101—H10C	109.5
C11—C13—H13C	109.5	H10B—C101—H10C	109.5
H13A—C13—H13C	109.5	N101—C102—C103	179.8 (9)
H13B—C13—H13C	109.5	C102—C103—H10D	109.5
C11—C14—H14A	109.5	C102—C103—H10E	109.5
C11—C14—H14B	109.5	H10D—C103—H10E	109.5
H14A—C14—H14B	109.5	C102—C103—H10F	109.5
C11—C14—H14C	109.5	H10D—C103—H10F	109.5
H14A—C14—H14C	109.5	H10E—C103—H10F	109.5
H14B—C14—H14C	109.5	N102—C104—C105	177.4 (12)
C16—C15—C8	109.6 (6)	C104—C105—H10G	109.5
C16—C15—C17	113.0 (7)	C104—C105—H10H	109.5
C8—C15—C17	108.0 (5)	H10G—C105—H10H	109.5
C16—C15—C18	105.6 (8)	C104—C105—H10I	109.5
C8—C15—C18	112.0 (6)	H10G—C105—H10I	109.5
C17—C15—C18	108.7 (7)	H10H—C105—H10I	109.5
C15—C16—H16A	109.5	N103—C106—C107	179.2 (9)
C15—C16—H16B	109.5	C106—C107—H10J	109.5
H16A—C16—H16B	109.5	C106—C107—H10K	109.5
C15—C16—H16C	109.5	H10J—C107—H10K	109.5
H16A—C16—H16C	109.5	C106—C107—H10L	109.5
H16B—C16—H16C	109.5	H10J—C107—H10L	109.5
C15—C17—H17A	109.5	H10K—C107—H10L	109.5
C15—C17—H17B	109.5		
			
O10—Mo4—O1—Mo2	−175.17 (17)	N4—Mo1—N2—C10	3.5 (3)
O9—Mo4—O1—Mo2	−69.85 (18)	N1—Mo1—N2—C10	177.1 (3)
O7^i^—Mo4—O1—Mo2	19.3 (4)	N5—Mo1—N2—C10	−127.3 (6)
O3—Mo4—O1—Mo2	84.09 (17)	N3—Mo1—N2—C6	−94.4 (3)
O4—Mo4—O1—Mo2	7.94 (13)	N6—Mo1—N2—C6	94.8 (3)
O6—Mo2—O1—Mo4	173.60 (19)	N4—Mo1—N2—C6	−171.3 (3)
O5—Mo2—O1—Mo4	70.10 (17)	N1—Mo1—N2—C6	2.3 (2)
O7—Mo2—O1—Mo4	−21.0 (4)	N5—Mo1—N2—C6	57.9 (7)
O2—Mo2—O1—Mo4	−84.47 (17)	N6—Mo1—N3—C19	−142.9 (5)
O4—Mo2—O1—Mo4	−7.91 (13)	N2—Mo1—N3—C19	85.9 (3)
O8—Mo3—O2—Mo2	178.69 (18)	N4—Mo1—N3—C19	−179.9 (3)
O3—Mo3—O2—Mo2	−74.98 (18)	N1—Mo1—N3—C19	8.3 (3)
O9^i^—Mo3—O2—Mo2	77.07 (17)	N5—Mo1—N3—C19	−89.2 (3)
O5^i^—Mo3—O2—Mo2	11.6 (4)	N6—Mo1—N3—C23	38.4 (7)
O4—Mo3—O2—Mo2	1.33 (13)	N2—Mo1—N3—C23	−92.7 (3)
O6—Mo2—O2—Mo3	175.20 (18)	N4—Mo1—N3—C23	1.4 (2)
O5—Mo2—O2—Mo3	5.4 (4)	N1—Mo1—N3—C23	−170.3 (3)
O7—Mo2—O2—Mo3	−79.44 (18)	N5—Mo1—N3—C23	92.1 (3)
O1—Mo2—O2—Mo3	75.27 (18)	N3—Mo1—N4—C28	178.7 (3)
O4—Mo2—O2—Mo3	−1.35 (13)	N6—Mo1—N4—C28	5.9 (3)
O8—Mo3—O3—Mo4	176.90 (18)	N2—Mo1—N4—C28	−89.8 (3)
O2—Mo3—O3—Mo4	70.14 (17)	N1—Mo1—N4—C28	−127.7 (7)
O9^i^—Mo3—O3—Mo4	−20.1 (4)	N5—Mo1—N4—C28	82.4 (3)
O5^i^—Mo3—O3—Mo4	−82.65 (16)	N3—Mo1—N4—C24	−1.0 (2)
O4—Mo3—O3—Mo4	−6.87 (12)	N6—Mo1—N4—C24	−173.7 (3)
O10—Mo4—O3—Mo3	−172.48 (17)	N2—Mo1—N4—C24	90.6 (3)
O9—Mo4—O3—Mo3	15.8 (4)	N1—Mo1—N4—C24	52.6 (8)
O1—Mo4—O3—Mo3	−70.70 (17)	N5—Mo1—N4—C24	−97.3 (3)
O7^i^—Mo4—O3—Mo3	84.58 (17)	N3—Mo1—N5—C37	10.9 (4)
O4—Mo4—O3—Mo3	6.99 (12)	N6—Mo1—N5—C37	−179.1 (4)
O9—Mo4—O4—Mo3	178.90 (9)	N2—Mo1—N5—C37	−141.2 (6)
O1—Mo4—O4—Mo3	84.15 (9)	N4—Mo1—N5—C37	87.9 (3)
O7^i^—Mo4—O4—Mo3	−90.53 (9)	N1—Mo1—N5—C37	−87.0 (3)
O9—Mo4—O4—Mo3^i^	−1.10 (9)	N3—Mo1—N5—C41	−158.5 (3)
O1—Mo4—O4—Mo3^i^	−95.85 (9)	N6—Mo1—N5—C41	11.5 (3)
O7^i^—Mo4—O4—Mo3^i^	89.47 (9)	N2—Mo1—N5—C41	49.4 (8)
O9—Mo4—O4—Mo2	88.84 (9)	N4—Mo1—N5—C41	−81.6 (3)
O1—Mo4—O4—Mo2	−5.91 (9)	N1—Mo1—N5—C41	103.5 (3)
O7^i^—Mo4—O4—Mo2	179.42 (9)	N3—Mo1—N6—C46	−129.8 (5)
O9—Mo4—O4—Mo2^i^	−91.16 (9)	N2—Mo1—N6—C46	1.1 (3)
O1—Mo4—O4—Mo2^i^	174.09 (9)	N4—Mo1—N6—C46	−93.9 (3)
O7^i^—Mo4—O4—Mo2^i^	−0.58 (9)	N1—Mo1—N6—C46	78.8 (3)
O2—Mo3—O4—Mo4^i^	89.11 (10)	N5—Mo1—N6—C46	174.7 (3)
O3—Mo3—O4—Mo4^i^	−174.68 (9)	N3—Mo1—N6—C42	47.8 (7)
O9^i^—Mo3—O4—Mo4^i^	−1.04 (9)	N2—Mo1—N6—C42	178.7 (2)
O2—Mo3—O4—Mo4	−90.89 (10)	N4—Mo1—N6—C42	83.7 (3)
O3—Mo3—O4—Mo4	5.32 (9)	N1—Mo1—N6—C42	−103.6 (3)
O9^i^—Mo3—O4—Mo4	178.96 (9)	N5—Mo1—N6—C42	−7.7 (2)
O2—Mo3—O4—Mo2	−1.03 (10)	C5—N1—C1—C2	0.7 (6)
O3—Mo3—O4—Mo2	95.18 (9)	Mo1—N1—C1—C2	−173.4 (3)
O9^i^—Mo3—O4—Mo2	−91.18 (9)	N1—C1—C2—C3	−0.8 (6)
O2—Mo3—O4—Mo2^i^	178.97 (10)	C1—C2—C3—C4	−0.2 (6)
O3—Mo3—O4—Mo2^i^	−84.82 (9)	C1—C2—C3—C11	−178.7 (4)
O9^i^—Mo3—O4—Mo2^i^	88.82 (9)	C2—C3—C4—C5	1.4 (7)
O5—Mo2—O4—Mo4^i^	94.22 (10)	C11—C3—C4—C5	179.8 (5)
O7—Mo2—O4—Mo4^i^	−0.61 (10)	C1—N1—C5—C4	0.5 (5)
O1—Mo2—O4—Mo4^i^	−174.27 (9)	Mo1—N1—C5—C4	175.2 (3)
O5—Mo2—O4—Mo4	−85.78 (10)	C1—N1—C5—C6	−178.2 (3)
O7—Mo2—O4—Mo4	179.39 (10)	Mo1—N1—C5—C6	−3.6 (4)
O1—Mo2—O4—Mo4	5.73 (9)	C3—C4—C5—N1	−1.6 (6)
O5—Mo2—O4—Mo3	−175.90 (10)	C3—C4—C5—C6	177.1 (4)
O7—Mo2—O4—Mo3	89.27 (10)	C10—N2—C6—C7	0.7 (6)
O1—Mo2—O4—Mo3	−84.38 (9)	Mo1—N2—C6—C7	175.9 (3)
O5—Mo2—O4—Mo3^i^	4.10 (10)	C10—N2—C6—C5	179.8 (3)
O7—Mo2—O4—Mo3^i^	−90.73 (10)	Mo1—N2—C6—C5	−4.9 (4)
O1—Mo2—O4—Mo3^i^	95.62 (9)	N1—C5—C6—N2	5.7 (5)
O6—Mo2—O5—Mo3^i^	178.25 (17)	C4—C5—C6—N2	−173.1 (4)
O7—Mo2—O5—Mo3^i^	71.23 (17)	N1—C5—C6—C7	−175.2 (4)
O1—Mo2—O5—Mo3^i^	−80.35 (17)	C4—C5—C6—C7	6.1 (6)
O2—Mo2—O5—Mo3^i^	−12.0 (4)	N2—C6—C7—C8	0.6 (7)
O4—Mo2—O5—Mo3^i^	−5.30 (13)	C5—C6—C7—C8	−178.5 (4)
O6—Mo2—O7—Mo4^i^	178.86 (18)	C6—C7—C8—C9	−1.5 (7)
O5—Mo2—O7—Mo4^i^	−76.42 (17)	C6—C7—C8—C15	179.6 (5)
O1—Mo2—O7—Mo4^i^	13.8 (4)	C7—C8—C9—C10	1.2 (7)
O2—Mo2—O7—Mo4^i^	76.47 (17)	C15—C8—C9—C10	−180.0 (5)
O4—Mo2—O7—Mo4^i^	0.80 (12)	C6—N2—C10—C9	−1.0 (6)
O10—Mo4—O9—Mo3^i^	−178.89 (16)	Mo1—N2—C10—C9	−175.7 (3)
O1—Mo4—O9—Mo3^i^	77.84 (17)	C8—C9—C10—N2	0.1 (7)
O7^i^—Mo4—O9—Mo3^i^	−74.88 (16)	C2—C3—C11—C12	−46.1 (8)
O3—Mo4—O9—Mo3^i^	−7.2 (4)	C4—C3—C11—C12	135.5 (6)
O4—Mo4—O9—Mo3^i^	1.43 (12)	C2—C3—C11—C13	75.1 (8)
O18—Mo6—O11—Mo5	179.91 (18)	C4—C3—C11—C13	−103.3 (8)
O17—Mo6—O11—Mo5	73.55 (19)	C2—C3—C11—C14	−165.1 (6)
O13—Mo6—O11—Mo5	−78.32 (18)	C4—C3—C11—C14	16.6 (8)
O16^ii^—Mo6—O11—Mo5	−15.2 (4)	C9—C8—C15—C16	53.3 (8)
O14—Mo6—O11—Mo5	−2.60 (14)	C7—C8—C15—C16	−127.9 (7)
O15—Mo5—O11—Mo6	−175.15 (19)	C9—C8—C15—C17	−70.2 (8)
O16—Mo5—O11—Mo6	−2.6 (4)	C7—C8—C15—C17	108.6 (6)
O12—Mo5—O11—Mo6	80.47 (19)	C9—C8—C15—C18	170.1 (7)
O19^ii^—Mo5—O11—Mo6	−74.38 (18)	C7—C8—C15—C18	−11.1 (9)
O14—Mo5—O11—Mo6	2.63 (14)	C23—N3—C19—C20	−1.6 (6)
O15—Mo5—O12—Mo7	−177.97 (19)	Mo1—N3—C19—C20	179.7 (3)
O16—Mo5—O12—Mo7	77.7 (2)	N3—C19—C20—C21	1.6 (6)
O19^ii^—Mo5—O12—Mo7	−12.5 (4)	C19—C20—C21—C22	0.1 (6)
O11—Mo5—O12—Mo7	−75.92 (19)	C19—C20—C21—C29	−177.9 (4)
O14—Mo5—O12—Mo7	0.16 (14)	C20—C21—C22—C23	−1.6 (6)
O20—Mo7—O12—Mo5	−175.99 (18)	C29—C21—C22—C23	176.3 (4)
O13—Mo7—O12—Mo5	78.36 (19)	C19—N3—C23—C22	0.0 (5)
O19—Mo7—O12—Mo5	−13.9 (4)	Mo1—N3—C23—C22	178.8 (3)
O17^ii^—Mo7—O12—Mo5	−76.48 (19)	C19—N3—C23—C24	179.6 (3)
O14—Mo7—O12—Mo5	−0.17 (14)	Mo1—N3—C23—C24	−1.6 (4)
O20—Mo7—O13—Mo6	−177.71 (19)	C21—C22—C23—N3	1.6 (5)
O19—Mo7—O13—Mo6	78.92 (19)	C21—C22—C23—C24	−178.0 (3)
O12—Mo7—O13—Mo6	−73.36 (19)	C28—N4—C24—C25	1.4 (5)
O17^ii^—Mo7—O13—Mo6	−2.5 (4)	Mo1—N4—C24—C25	−178.9 (3)
O14—Mo7—O13—Mo6	2.61 (14)	C28—N4—C24—C23	−179.2 (3)
O18—Mo6—O13—Mo7	−179.0 (2)	Mo1—N4—C24—C23	0.5 (4)
O17—Mo6—O13—Mo7	−16.4 (4)	N3—C23—C24—N4	0.7 (4)
O11—Mo6—O13—Mo7	75.63 (19)	C22—C23—C24—N4	−179.7 (3)
O16^ii^—Mo6—O13—Mo7	−79.56 (19)	N3—C23—C24—C25	−179.9 (3)
O14—Mo6—O13—Mo7	−2.62 (14)	C22—C23—C24—C25	−0.3 (5)
O13—Mo7—O14—Mo5^ii^	88.07 (11)	N4—C24—C25—C26	−0.4 (5)
O19—Mo7—O14—Mo5^ii^	−6.40 (10)	C23—C24—C25—C26	−179.7 (3)
O12—Mo7—O14—Mo5^ii^	−179.88 (10)	C24—C25—C26—C27	−1.2 (5)
O17^ii^—Mo7—O14—Mo5^ii^	−94.30 (10)	C24—C25—C26—C33	179.5 (3)
O13—Mo7—O14—Mo5	−91.93 (11)	C25—C26—C27—C28	1.8 (6)
O19—Mo7—O14—Mo5	173.60 (10)	C33—C26—C27—C28	−178.9 (4)
O12—Mo7—O14—Mo5	0.12 (10)	C24—N4—C28—C27	−0.8 (6)
O17^ii^—Mo7—O14—Mo5	85.70 (10)	Mo1—N4—C28—C27	179.5 (3)
O13—Mo7—O14—Mo6^ii^	177.99 (11)	C26—C27—C28—N4	−0.8 (6)
O19—Mo7—O14—Mo6^ii^	83.51 (10)	C20—C21—C29—C32'	−124.0 (11)
O12—Mo7—O14—Mo6^ii^	−89.97 (10)	C22—C21—C29—C32'	58.1 (11)
O17^ii^—Mo7—O14—Mo6^ii^	−4.39 (10)	C20—C21—C29—C31	54.6 (6)
O13—Mo7—O14—Mo6	−2.01 (11)	C22—C21—C29—C31	−123.3 (5)
O19—Mo7—O14—Mo6	−96.49 (10)	C20—C21—C29—C32	−66.9 (6)
O12—Mo7—O14—Mo6	90.03 (10)	C22—C21—C29—C32	115.2 (5)
O17^ii^—Mo7—O14—Mo6	175.61 (10)	C20—C21—C29—C30'	111.9 (11)
O16—Mo5—O14—Mo7	−94.51 (11)	C22—C21—C29—C30'	−66.0 (11)
O12—Mo5—O14—Mo7	−0.12 (11)	C20—C21—C29—C30	174.5 (5)
O19^ii^—Mo5—O14—Mo7	173.75 (10)	C22—C21—C29—C30	−3.5 (6)
O11—Mo5—O14—Mo7	87.89 (10)	C20—C21—C29—C31'	−1.1 (12)
O16—Mo5—O14—Mo7^ii^	85.49 (11)	C22—C21—C29—C31'	−179.0 (11)
O12—Mo5—O14—Mo7^ii^	179.88 (11)	C27—C26—C33—C36'	76.5 (12)
O19^ii^—Mo5—O14—Mo7^ii^	−6.25 (10)	C25—C26—C33—C36'	−104.2 (12)
O11—Mo5—O14—Mo7^ii^	−92.11 (10)	C27—C26—C33—C35	−112.9 (5)
O16—Mo5—O14—Mo6^ii^	−4.27 (11)	C25—C26—C33—C35	66.4 (5)
O12—Mo5—O14—Mo6^ii^	90.11 (11)	C27—C26—C33—C35'	−58.4 (13)
O19^ii^—Mo5—O14—Mo6^ii^	−96.01 (10)	C25—C26—C33—C35'	120.9 (13)
O11—Mo5—O14—Mo6^ii^	178.13 (10)	C27—C26—C33—C34	123.5 (5)
O16—Mo5—O14—Mo6	175.73 (11)	C25—C26—C33—C34	−57.2 (5)
O12—Mo5—O14—Mo6	−89.89 (11)	C27—C26—C33—C36	7.4 (6)
O19^ii^—Mo5—O14—Mo6	83.99 (10)	C25—C26—C33—C36	−173.2 (4)
O11—Mo5—O14—Mo6	−1.87 (10)	C27—C26—C33—C34'	−163.9 (10)
O17—Mo6—O14—Mo7	175.28 (11)	C25—C26—C33—C34'	15.4 (11)
O11—Mo6—O14—Mo7	−88.07 (11)	C41—N5—C37—C38	0.3 (6)
O13—Mo6—O14—Mo7	1.89 (10)	Mo1—N5—C37—C38	−168.9 (3)
O16^ii^—Mo6—O14—Mo7	85.94 (10)	N5—C37—C38—C39	1.9 (7)
O17—Mo6—O14—Mo7^ii^	−4.72 (11)	C37—C38—C39—C40	−1.9 (6)
O11—Mo6—O14—Mo7^ii^	91.93 (11)	C37—C38—C39—C47	178.9 (4)
O13—Mo6—O14—Mo7^ii^	−178.11 (10)	C38—C39—C40—C41	−0.2 (6)
O16^ii^—Mo6—O14—Mo7^ii^	−94.06 (10)	C47—C39—C40—C41	179.0 (4)
O17—Mo6—O14—Mo5^ii^	85.35 (11)	C37—N5—C41—C40	−2.5 (6)
O11—Mo6—O14—Mo5^ii^	−178.00 (11)	Mo1—N5—C41—C40	167.8 (3)
O13—Mo6—O14—Mo5^ii^	−88.04 (10)	C37—N5—C41—C42	176.4 (3)
O16^ii^—Mo6—O14—Mo5^ii^	−3.99 (10)	Mo1—N5—C41—C42	−13.3 (4)
O17—Mo6—O14—Mo5	−94.65 (11)	C39—C40—C41—N5	2.5 (6)
O11—Mo6—O14—Mo5	2.00 (11)	C39—C40—C41—C42	−176.3 (3)
O13—Mo6—O14—Mo5	91.96 (10)	C46—N6—C42—C43	−0.4 (5)
O16^ii^—Mo6—O14—Mo5	176.01 (10)	Mo1—N6—C42—C43	−178.2 (3)
O15—Mo5—O16—Mo6^ii^	−176.79 (19)	C46—N6—C42—C41	−179.0 (3)
O12—Mo5—O16—Mo6^ii^	−70.82 (19)	Mo1—N6—C42—C41	3.3 (4)
O19^ii^—Mo5—O16—Mo6^ii^	81.06 (19)	N5—C41—C42—N6	6.7 (5)
O11—Mo5—O16—Mo6^ii^	10.7 (4)	C40—C41—C42—N6	−174.4 (3)
O14—Mo5—O16—Mo6^ii^	5.51 (14)	N5—C41—C42—C43	−171.8 (3)
O18—Mo6—O17—Mo7^ii^	−177.81 (19)	C40—C41—C42—C43	7.1 (6)
O11—Mo6—O17—Mo7^ii^	−70.56 (19)	N6—C42—C43—C44	−0.4 (5)
O13—Mo6—O17—Mo7^ii^	19.8 (4)	C41—C42—C43—C44	178.0 (3)
O16^ii^—Mo6—O17—Mo7^ii^	81.73 (19)	C42—C43—C44—C45	0.9 (5)
O14—Mo6—O17—Mo7^ii^	6.11 (14)	C42—C43—C44—C51	178.4 (3)
O20—Mo7—O19—Mo5^ii^	−175.57 (19)	C43—C44—C45—C46	−0.5 (5)
O13—Mo7—O19—Mo5^ii^	−69.43 (19)	C51—C44—C45—C46	−178.0 (3)
O12—Mo7—O19—Mo5^ii^	22.2 (4)	C42—N6—C46—C45	0.8 (5)
O17^ii^—Mo7—O19—Mo5^ii^	84.27 (18)	Mo1—N6—C46—C45	178.3 (3)
O14—Mo7—O19—Mo5^ii^	8.53 (14)	C44—C45—C46—N6	−0.3 (6)
N3—Mo1—N1—C5	91.9 (3)	C38—C39—C47—C50	120.2 (7)
N6—Mo1—N1—C5	−93.9 (3)	C40—C39—C47—C50	−58.9 (7)
N2—Mo1—N1—C5	0.8 (2)	C38—C39—C47—C48	−5.2 (7)
N4—Mo1—N1—C5	39.7 (8)	C40—C39—C47—C48	175.7 (5)
N5—Mo1—N1—C5	−170.6 (3)	C38—C39—C47—C49	−124.0 (5)
N3—Mo1—N1—C1	−93.9 (3)	C40—C39—C47—C49	56.9 (6)
N6—Mo1—N1—C1	80.2 (3)	C45—C44—C51—C52	111.0 (4)
N2—Mo1—N1—C1	175.0 (3)	C43—C44—C51—C52	−66.4 (5)
N4—Mo1—N1—C1	−146.1 (6)	C45—C44—C51—C54	−9.0 (6)
N5—Mo1—N1—C1	3.6 (3)	C43—C44—C51—C54	173.6 (4)
N3—Mo1—N2—C10	80.4 (3)	C45—C44—C51—C53	−129.3 (4)
N6—Mo1—N2—C10	−90.4 (3)	C43—C44—C51—C53	53.3 (5)

Symmetry codes: (i) −*x*+1, −*y*+2, −*z*+1; (ii) −*x*+1, −*y*+1, −*z*+2.

### Table 2 Selected short distance interactions (Å, °)

**Table:**

*A*—*B*···*C*	*A*—*B*	*B*···*C*	*A*···*C*	<(*A*—*B*···*C*)
*Y*—*X*···π contacts				
Mo4—O10···Cg1^i^	1.690 (3)	3.151 (4)	4.393 (2)	127.74 (15)
Mo5—O15···Cg2^ii^	1.686 (3)	3.399 (5)	4.622 (2)	127.5 (2)
C102—N101···Cg2	1.160 (10)	3.395 (9)	3.473 (8)	84.1 (6)
C102—N101···Cg3	1.160 (10)	3.558 (8)	3.762 (8)	91.1 (6)
Weak hydrogen bonds				
C16—H16A···N101^ii^	0.98	2.60	3.537 (14)	160
C19—H19···O10^i^	0.95	2.45	3.331 (5)	154
C27—H27···O17^ii^	0.95	2.57	3.059 (6)	113
C36—H36A···O8^iii^	0.98	2.55	3.501 (8)	164
C49—H49C···O6^iv^	0.98	2.59	3.557 (8)	170

Symmetry codes: (i) 1-*x*, 1-*y*, 1-*z*; (ii) 2-*x*, 1-*y*, 2-*z*; (iii) 1+*x*, -1+*y*, 1+*z*; (iv) 2-*x*, 1-*y*, 1-*z*. Cg1: centroid of the ring formed by C1 to C5; Cg2: centroid of the ring formed by C6 to C10; Cg3: centroid of the ring formed by C19 to C23.
